# Activity-Based Cell Sorting Reveals Resistance of Functionally Degenerate *Nitrospira* during a Press Disturbance in Nitrifying Activated Sludge

**DOI:** 10.1128/mSystems.00712-21

**Published:** 2021-07-20

**Authors:** Maxwell B. W. Madill, Yaqian Luo, Pranav Sampara, Ryan M. Ziels

**Affiliations:** a Department of Civil Engineering, The University of British Columbia, Vancouver, Canada; University of California, San Diego

**Keywords:** BONCAT, activated sludge, cell sorting, ecophysiology, functional degeneracy, nitrification, nitrite oxidation, translation, wastewater treatment

## Abstract

Managing and engineering activated sludge wastewater treatment microbiomes for low-energy nitrogen removal requires process control strategies to stop the oxidation of ammonium at nitrite. Our ability to out-select nitrite-oxidizing bacteria (NOB) from activated sludge is challenged by their metabolic and physiological diversity, warranting measurements of their *in situ* physiology and activity under selective growth pressures. Here, we examined the stability of nitrite oxidation in activated sludge during a press disturbance induced by treating a portion of return activated sludge with a sidestream flow containing free ammonia (FA) at 200 mg NH_3_-N/liter. The nitrite accumulation ratio peaked at 42% by day 40 in the experimental bioreactor with the press disturbance, while it did not increase in the control bioreactor. A subsequent decrease in nitrite accumulation within the experimental bioreactor coincided with shifts in dominant *Nitrospira* 16S rRNA amplicon sequence variants (ASVs). We applied bioorthogonal noncanonical amino acid tagging (BONCAT) coupled with fluorescence-activated cell sorting (FACS) to investigate changes in the translational activity of NOB populations throughout batch exposure to FA. BONCAT-FACS confirmed that the single *Nitrospira* ASV washed out of the experimental bioreactor had reduced translational activity following exposure to FA, whereas the two *Nitrospira* ASVs that emerged after process acclimation were not impacted by FA. Thus, the coexistence of functionally degenerate and physiologically resistant *Nitrospira* populations provided resilience to the nitrite-oxidizing function during the press disturbance. These results highlight how BONCAT-FACS can resolve ecological niche differentiation within activated sludge and inform strategies to engineer and control microbiome function.

**IMPORTANCE** Nitrogen removal from activated sludge wastewater treatment systems is an energy-intensive process due to the large aeration requirement for nitrification. This energy footprint could be minimized with engineering control strategies that wash out nitrite-oxidizing bacteria (NOB) to limit oxygen demands. However, NOB populations can have a high degree of physiological diversity, and it is currently difficult to decipher the behavior of individual taxa during applied selective pressures. Here, we utilized a new substrate analog probing approach to measure the activity of NOB at the cellular translational level in the face of a press disturbance applied to the activated sludge process. Substrate analog probing corroborated the time series reactor sampling, showing that coexisting and functionally degenerate *Nitrospira* populations provided resilience to the nitrite oxidation process. Taken together, these results highlight how substrate analog approaches can illuminate *in situ* ecophysiologies within shared niches, and can inform strategies to improve microbiome engineering and management.

## INTRODUCTION

Relating the *in situ* physiological responses of individual taxa in the face of environmental perturbations to the resulting microbial community structure and function remains a critical challenge for controlling and engineering microbiomes for desirable ecological processes and outcomes (1–3). Biological wastewater treatment processes are ideal ecosystems to explore such relationships, as environmental conditions can be manipulated, and the community function can be monitored, in a relatively controlled manner (4). A major goal in the wastewater industry is to engineer microbial bioprocesses to achieve energy-efficient, or even net energy-positive, wastewater treatment (5). A foundational component of achieving this goal is the optimization of mainstream biological nitrogen removal processes, as conventionally, this process is the largest consumer of energy and exogenous organic carbon within wastewater treatment plants (WWTPs) (6, 7). Realizing energy-efficient nitrogen removal requires highly finessed and sustained modulation of the abundances, activities, and interactions of key microbial functional groups to effectively control the global community function and engage the desired nitrogen removal pathway(s) (8, 9). As such pathways typically impose inherent energetic and/or metabolic constraints (10, 11), and often challenge existing community interactions (8, 9), it is critical to fully illuminate the ecophysiological diversity and mechanisms driving niche partitioning within these microbial functional groups as well as their responses to the applied process control strategies (9).

An appealing strategy to achieve energy-efficient biological nitrogen removal is to limit the nitrification process to nitritation (i.e., oxidation of ammonium to nitrite), as the resulting nitrite can be denitrified directly (25% and 40% net energy and carbon reductions, respectively) and/or provided to anammox bacteria as a growth substrate for autotrophic nitrogen removal (60% and 100% net energy and carbon reductions, respectively) (8, 12). However, achieving stable nitritation in mainstream activated sludge (AS) stands as a major challenge limiting the successful full-scale implementation of these energy-efficient removal processes (13–15). Realizing stable nitritation in mainstream AS relies on engineering control strategies that serve as press disturbances to consistently repress and wash out nitrite-oxidizing bacteria (NOB) while maintaining the activity of ammonium-oxidizing bacteria (AOB) (8, 9, 13). The efficacy of a given control strategy is therefore dependent on its ability to create a disturbance that elicits distinct physiological responses between AOB and NOB. Preliminary success in washing out NOB from mainstream AS has been achieved using press disturbances that provide a high ammonium residual (13), or control the availability of dissolved oxygen (DO) (13, 16, 17), to favor the growth kinetics of AOB over NOB. Additionally, several recently proposed control strategies have utilized the higher innate sensitivity of NOB to free ammonia (FA) and free nitrous acid (FNA) compared to AOB (18–20) to achieve effective NOB inhibition (21–23). Wang et al. (21) demonstrated that a press disturbance induced by exposing a fraction of return sludge to FA-rich sidestream wastewater (210 mg NH_3_-N/liter) supported successful NOB washout in mainstream AS, with nitrite accumulation ratios (NARs) reaching 80 to 90%. Despite its potential efficacy for supporting mainstream nitritation, there have been a limited number of studies evaluating the role of niche differentiation and physiological diversity in the stability of nitrite oxidation in the face of a press disturbance from routine FA exposure.

NOB communities in wastewater treatment often display functional degeneracy, wherein the nitrite oxidation process is distributed among several phylogenetically diverse taxa with various auxiliary metabolic potentials (24–29). Inherent differences in nitrite oxidation biochemistry and cell morphology play key roles in supporting ecophysiological diversity between NOB genera by influencing their substrate affinities for oxygen and nitrite, and their nitrite oxidation kinetics (24, 30, 31). *Nitrospira*, a predominant NOB genus in many WWTP microbiomes (32–34), has demonstrated an extraordinary degree of functional degeneracy, with reports of highly complex and stable communities containing as many as 120 closely related coexisting strains (26, 27, 34). Considerable ecophysiological diversity may thus exist between *Nitrospira* species/strains to support niche partitioning, which could be supported by their distinct oxygen and nitrite preferences (34–36), auxiliary metabolic potentials for utilizing alternative electron acceptors and/or donors (24, 27, 34, 37, 38), and tolerances to challenging environmental conditions, including FA (27, 28, 39, 40). Exhibited at both the genus and strain levels, such functional degeneracy may enable NOB communities to resist the selective pressures imparted by engineering process control strategies by recruiting functionally redundant, yet physiologically diverse, NOB members (41–43). *In situ* assessments of the metabolically active fraction of nitrifying communities are therefore critical to evaluate the efficacy of mainstream nitritation control strategies and elucidate their associated impacts on functionally degenerate NOB.

Next-generation substrate analog probing (SAP) approaches have recently emerged as powerful tools to decipher the *in situ* physiology of active cells based on their uptake of synthetic analogs of natural biomolecules (44). Bioorthogonal noncanonical amino acid tagging (BONCAT) is a nascent SAP approach to study the physiology of active cells in complex environmental microbiomes (45–48). BONCAT relies on the *in vivo* uptake and incorporation of synthetic amino acids, such as the alkyne-containing analog of methionine, homopropargylglycine (HPG), into newly synthesized proteins via the native translational machinery and thereby selectively labels the proteomes of translationally active cells (44, 47). HPG-labeled cells can subsequently be identified by tagging their proteins with azide-modified fluorescent dyes via azide-alkyne click chemistry, enabling their selective recovery using fluorescence-activated cell sorting (FACS) (45, 46, 48). To our knowledge, BONCAT, or its paired approach with FACS (BONCAT-FACS), has yet to be applied to study the active fractions of AS microbiomes central to wastewater treatment bioprocesses.

The objective of this study was to assess the stability of nitrite oxidation in the face of a press disturbance induced by routine exposure of return activated sludge to FA as an engineering control strategy to wash out NOB. We hypothesized that certain members of the active NOB microbial community could acclimate to the applied press disturbance. Two parallel experimental and control AS sequencing batch reactors (SBRs) were operated for ∼100 days to investigate the impacts of routine FA exposure as a press disturbance on the NOB community. We applied time series 16S rRNA gene amplicon sequencing in addition to BONCAT-FACS-based activity measurements to elucidate changes in the structure and *in situ* activity of the AS microbiome and nitrifying communities.

## RESULTS

### Partial nitritation performance of activated sludge bioreactors.

**FIG 1:**
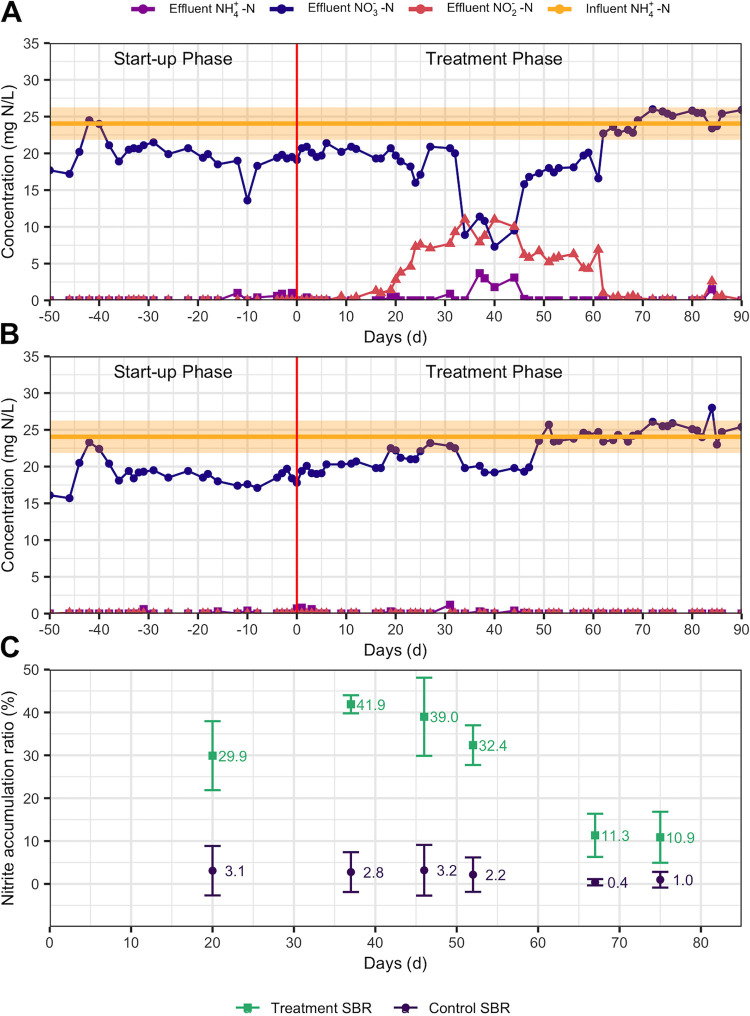
(A and B) Influent and effluent nitrogen compounds over the two experimental phases in the treatment sequencing batch reactor (SBR) (A) and the control SBR (B). The shaded orange space represents the standard deviation of influent NH_4_^+^-N. (C) Nitrite accumulation ratios (NARs) (percent) of treatment and control SBRs over time, based on 24-h monitoring of SBR effluents. NAR is defined as the effluent nitrite (milligrams of N per liter) divided by the effluent nitrite plus nitrate (milligrams of N per liter) and indicates the level of NOB activity suppression.

Two AS SBRs treated synthetic mainstream municipal wastewater over two operational phases: the start-up phase and the treatment phase (see Fig. S1 in the supplemental material). Periodic steady-state conditions were presumed over the last 30 days of the 270-day start-up phase, during which there were no significant differences in daily effluent concentrations of NH_4_^+^-N, NO_3_^−^-N, and NO_2_^−^-N between the two SBRs (*P > *0.05), which averaged 0.1 ± 0.3 mg NH_4_^+^-N/liter, 19 ± 2 mg NO_3_^−^-N/liter, and 0.0 ± 0.01 mg NO_2_^−^-N/liter, respectively (Fig. 1). There were also no significant differences in the total suspended solids (TSS) and volatile suspended solids (VSS) of mixed liquor between the two systems during the start-up phase (*P* > 0.05) (see Fig. S2 at https://doi.org/10.6084/m9.figshare.14787984). Thus, similar stable performances with full nitrification were achieved in both SBRs during the initial start-up phase, indicating effective duplication of operating conditions.

10.1128/mSystems.00712-21.2 FIG S1 Schematic diagrams showing the operation of the two SBRs during the start-up phase (A) and the treatment phase (B). ER, experimental reactor (also termed treatment SBR); CR, control SBR; FA, free ammonia (milligrams of NH_3_-N per liter); Q_0_, feed flow rate of synthetic wastewater; Q_E_, effluent flow rate; Q_W_, waste activated sludge flow rate; Q_r_, sidestream return sludge treatment flow rate. Download FIG S1, PDF file, 0.2 MB. Copyright © 2021 Madill et al. 2021 Madill et al. https://creativecommons.org/licenses/by/4.0/ This content is distributed under the terms of the Creative Commons Attribution 4.0 International license.

The treatment phase was then commenced on day 0 to assess the impacts of a press disturbance induced by routine FA exposure of return sludge in a sidestream reactor on the nitrifying community structure and activity. Approximately 20% of the return sludge in the treatment SBR was exposed to 200 mg NH_3_-N/liter as FA at a pH of 9.0 for 24 h before being reintroduced into the mainstream SBR on a daily basis, while the same conditions were emulated for the control SBR but without FA added to the sidestream. Additionally, ammonium nitrogen was added (160 mg NH_4_^+^-N/day) to the control SBR on a daily basis along with the return sludge to maintain equivalent nitrogen loadings to the two SBRs. After 10 days of the applied press disturbance, NO_2_^−^-N began to increase in the effluent of the treatment SBR but stayed below the detection level in the control SBR for the remainder of the treatment phase (Fig. 1). By day 40, effluent NO_2_^−^-N reached its peak level of 11 mg NO_2_^−^-N/liter in the treatment SBR. At the same time, NH_4_^+^-N accumulated to 3.7 mg NH_4_^+^-N/liter in the treatment SBR between days 37 and 44, yet stayed below 1.2 mg NH_4_^+^-N/liter in the control SBR over the entire treatment phase (Fig. 1). The accumulation of NO_2_^−^-N in the treatment SBR was transient, however, as the effluent concentration decreased after day 40 and reached a value below the detection level by day 74 (Fig. 1).

As effluent was sampled from the SBRs on a 24-h basis, and the sidestream return sludge was added once every 24 h, periodic tests were conducted to measure nitrogen compounds at the end of individual SBR cycles over the course of 24 h to better estimate NARs (Fig. S3). After 20 days of the press disturbance, the 24-h average NAR was approximately 10 times higher in the treatment SBR than in the control SBR (Fig. 1C). The NAR reached its peak of 41.9% by day 37 in the treatment SBR, aligning with the observed peak in effluent NO_2_^−^-N concentrations. After day 37, the NAR decreased in the treatment SBR, reaching its lowest observed level of 10.9% on day 75. The NAR of the control SBR stayed below 3.2% for the entire treatment phase (Fig. 1C). This suggests that the nitrite oxidation function was resilient to the press disturbance of routine FA exposure, as the extent of nitrite oxidation inhibition was not sustained after about 40 days.

10.1128/mSystems.00712-21.3 FIG S3 Ammonium (NH_4_^+^-N), nitrite (NO_2_^−^-N), and nitrate (NO_3_^−^-N) concentrations in the treatment SBR and the control SBR throughout a 24-h period, measured on day 37 (A) and day 75 (B) of the treatment phase. The timing of the SBR cycles is marked with vertical dashed lines. Download FIG S3, PDF file, 0.4 MB. Copyright © 2021 Madill et al. 2021 Madill et al. https://creativecommons.org/licenses/by/4.0/ This content is distributed under the terms of the Creative Commons Attribution 4.0 International license.

### Microbial community acclimation to routine FA exposure.

**FIG 2:**
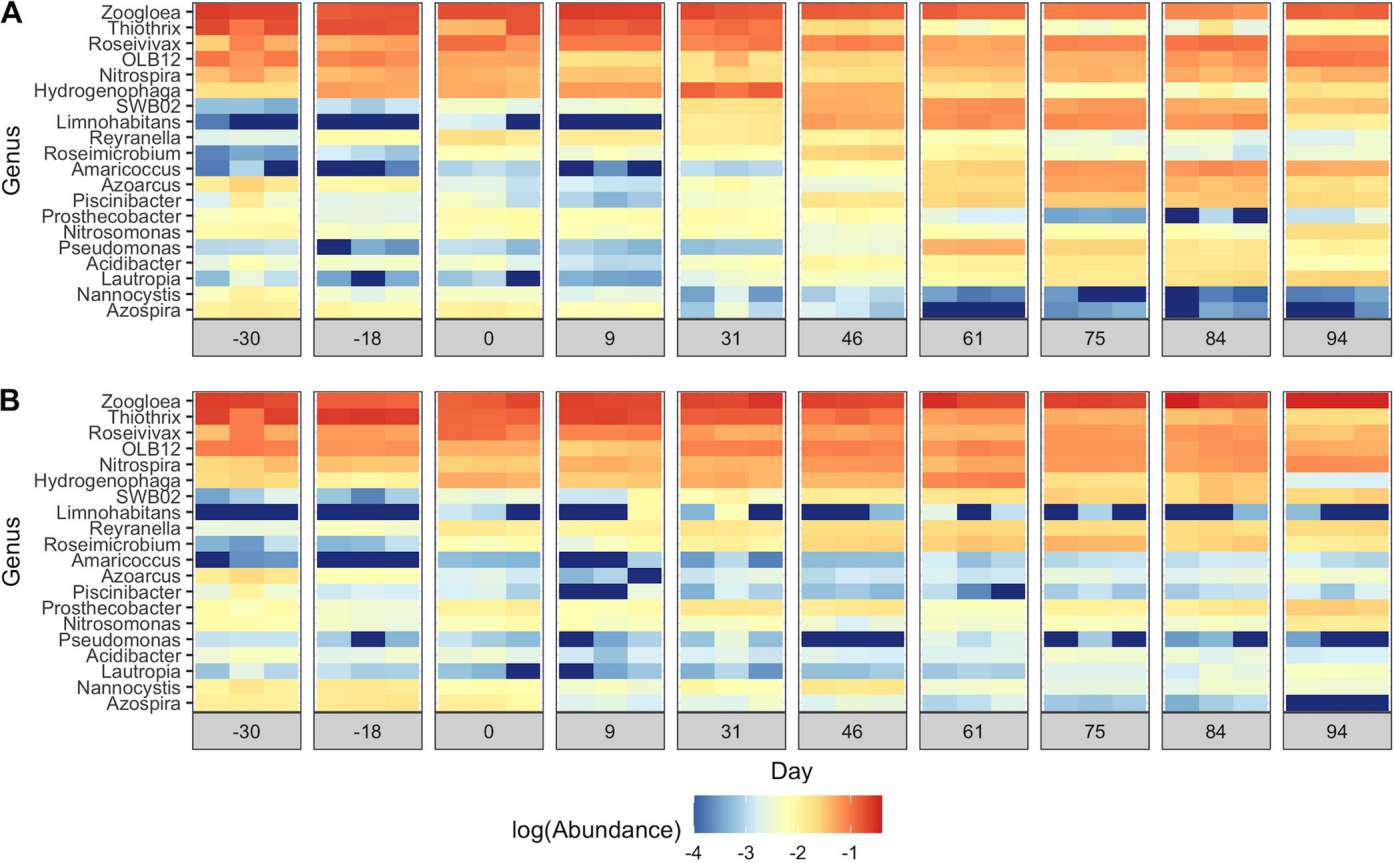
Heat map of log-scaled relative abundances of the top 20 most abundant genera in the treatment SBR (A) and the control SBR (B) over the two operational phases. Sequencing results are shown for triplicate DNA extractions on each sampling date. The genera are ordered from highest to lowest cumulative abundances across samples.

Microbial 16S rRNA gene amplicons were denoised into amplicon sequence variants (ASVs) to provide a high-resolution (49, 50) view of how routine FA exposure impacted the community structure. A total of 3.01 million chimera-free quality-filtered merged reads were denoised into 6,694 ASVs. Over 95% of the 16S rRNA gene amplicons at all time points in both SBRs were comprised of the 8 phyla: *Proteobacteria*, *Bacteroidetes*, *Chloroflexi*, *Nitrospirae*, *Planctomycetes*, *Verrucomicrobia*, *Acidobacteria*, and *Cyanobacteria* (see Fig. S4 at https://doi.org/10.6084/m9.figshare.14787984). Between 53% and 70% of 16S rRNA amplicons were represented by 20 genera across all samples (Fig. 2). Even at the broad genus level of resolution, there were apparent differences in community profiles between the treatment and control SBRs over time (Fig. 2), indicating that routine FA exposure altered the structure of the AS microbiome.

**FIG 3:**
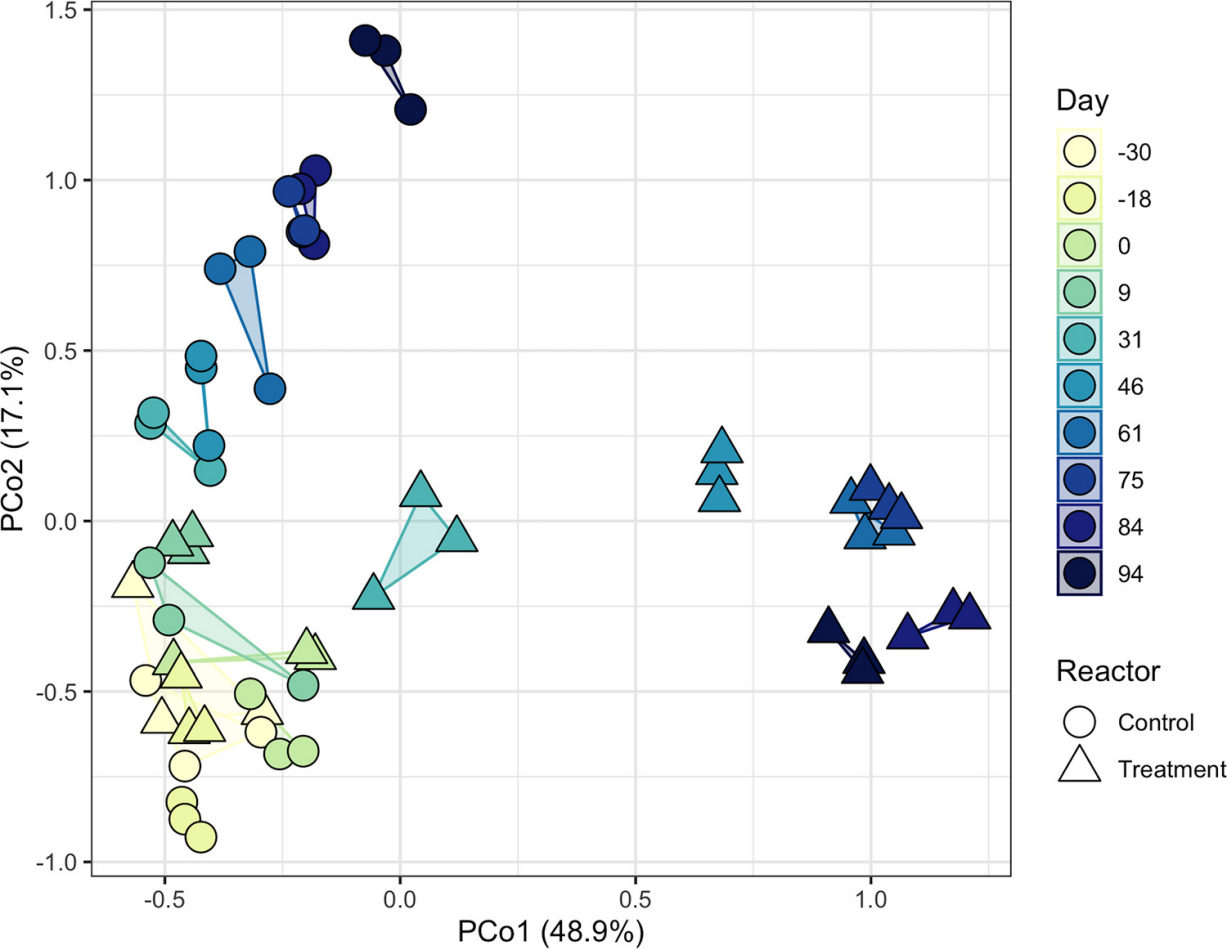
Principal coordinate analysis (PCoA) of Bray-Curtis dissimilarities between cumulative sum-scaled (CSS) read counts of 16S rRNA ASVs in both SBRs over time. The marker fill represents the reactor operating day, and the marker shape corresponds to the SBR. Triplicate DNA extractions for each time point are indicated by a shared polygon. The percentages in parentheses represent the fraction of the variance explained by that axis.

At the ASV level, FA exposure of return sludge led to significant differences in community structure between the two SBRs over time (*R*^2^ = 0.54; *P < *0.001 [adonis]). Principal coordinate analysis (PCoA) of cumulative sum-scaled (CSS) ASV read counts revealed that the community profiles of the two SBRs were similar until day 9, after which the treatment SBR community diverged from the control (Fig. 3). Differential abundance analysis showed that there were no statistically different ASVs between the two SBRs on day 0 (*P > *0.01 [DESeq2]), indicating that they were well replicated in the start-up phase. By day 46 of the treatment phase, around the time when nitrite peaked in the treatment SBR (Fig. 1), 105 ASVs spanning 55 genera were differentially abundant between the two SBRs (*P < *0.01 [DESeq2]) (see Fig. S5 at https://doi.org/10.6084/m9.figshare.14787984). The number of ASVs with significant differential abundances between the SBRs continued to increase to a maximum of 166 on day 75 of the treatment phase (Fig. S5).

10.1128/mSystems.00712-21.6 FIG S8 BONCAT-FACS results generated from preliminary validation microcosms in which cells were incubated without HPG (A), fixed with 3% paraformaldehyde before incubation with HPG (B), and fixed with 3% paraformaldehyde after incubation with HPG (C) (see Text S1 in the supplemental material). The gating area shows STYO^+^ and BONCAT^+^ cell fluorescence determinations based on a 0.5% false-positive rate. The fractional abundance of BONCAT^+^ cells in each sample was calculated as the fraction of SYTO^+^ cells residing in the sorting gate, as indicated in red text in each box. The absence of BONCAT^+^ cells in the pre-incubation fixed control samples (B) but the detection of BONCAT^+^ cells in the post-incubation fixed samples (C) indicates that cells needed to actively take up and incorporate HPG to become fluorescently labeled by the FAM picolyl azide BONCAT dye. The lower rate of detection of BONCAT^+^ cells within the post-incubation fixed samples may be attributed to a potentially lower level of translational activity within the control SBR mixed liquor during the time of conducting the validation microcosms or to potential impacts of cellular fixation on sample homogenization and/or click chemistry labeling efficiencies. Download FIG S8, PDF file, 1.2 MB. Copyright © 2021 Madill et al. 2021 Madill et al. https://creativecommons.org/licenses/by/4.0/ This content is distributed under the terms of the Creative Commons Attribution 4.0 International license.

10.1128/mSystems.00712-21.1 TEXT S1 Supplemental methods. Download Text S1, PDF file, 0.2 MB. Copyright © 2021 Madill et al. 2021 Madill et al. https://creativecommons.org/licenses/by/4.0/ This content is distributed under the terms of the Creative Commons Attribution 4.0 International license.

**FIG 4:**
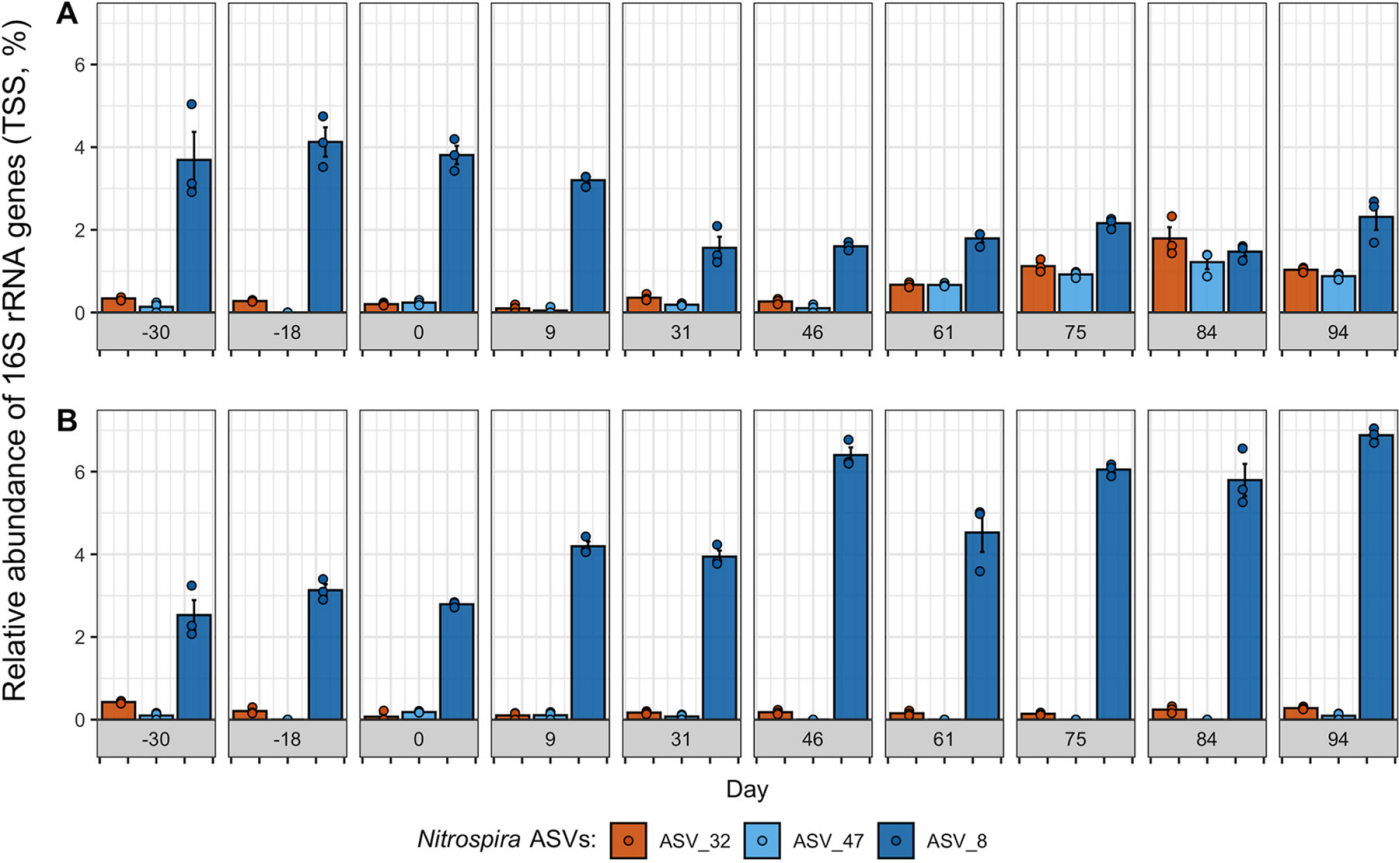
Relative abundance (total sum scaled [TSS]) of three dominant *Nitrospira* ASVs over time in the treatment SBR (A) and the control SBR (B). Only *Nitrospira* ASVs detected in more than one sample are shown. Results are shown for triplicate DNA extractions on each day, with the colored points showing the relative abundance of each ASV in each DNA extraction, the colored bar showing the mean relative abundance, and the error bars showing the standard errors of the means.

*Nitrospira* and *Nitrosomonas* were the only putative NOB and AOB populations detected in the SBRs, respectively (Fig. 2). As the reactors were fed with synthetic wastewater, it is likely that these populations originated from the inoculum. Six dominant *Nitrosomonas* ASVs were detected in both SBRs over the two experimental phases (Fig. S6). Until day 84, the total *Nitrosomonas* abundance was less than 1% in both SBRs but increased to over 1.5% in both SBRs by day 94 (Fig. S6). One *Nitrosomonas* ASV (ASV_36) was differentially abundant between the two SBRs on days 75 and 94 (*P* < 0.01 [DESeq2]). Three *Nitrospira* ASVs were detected within the two SBRs (Fig. 4). In particular, ASV_8 was the dominant *Nitrospira* ASV in both SBRs during the start-up phase (before day 0), accounting for 3.3% ± 0.8% of 16S rRNA genes on average (Fig. 4). By day 46, ASV_8 decreased to 1.6% ± 0.1% in the treatment SBR, while it increased to 6.4% ± 0.3% within the control. This decrease in ASV_8 abundance coincided with the peak in nitrite accumulation in the treatment SBR (Fig. 1A). The abundance of ASV_8 did not significantly change after day 46 for the remainder of the experiment in the treatment SBR (*P* > 0.01 [DESeq2]). In contrast, two other *Nitrospira* ASVs (ASV_32 and ASV_47 [99.7% sequence similarity to each other; 94.2% and 94.5% sequence similarities to ASV_8, respectively]) were sporadically detected in both SBRs at abundances below 0.4% until day 46 and then increased to maximum values of 1.8% ± 0.4% and 1.2% ± 0.3% in the treatment SBR by day 84, respectively, but stayed below 0.3% in the control (Fig. 4). The abundances of ASV_32 and ASV_47 were significantly higher in the treatment SBR than in the control SBR by the end of the experiment, while the abundance of ASV_8 was significantly lower (both days 84 and 94) (*P < *0.01 [DESeq2]). Phylogenetic analysis based on partial 16S rRNA gene sequences (Fig. S7) revealed that ASV_8 was most closely related to Nitrospira lenta within lineage II, whereas ASV_32 and ASV_47 were clustered within lineage I of *Nitrospira* (51, 52).

10.1128/mSystems.00712-21.4 FIG S6 Relative abundances (total sum scaled [TSS]) of 6 dominant *Nitrosomonas* ASVs over time in the treatment SBR (A) and the control SBR (B). Only *Nitrosomonas* ASVs with mean relative abundances of >0.05% detected in at least one sample are shown. Results are shown for triplicate DNA extractions on each day, with the colored points showing the relative abundance of each ASV in each DNA extraction, the colored bar showing the mean relative abundance, and the error bars showing the standard errors of the means. Download FIG S6, PDF file, 0.5 MB. Copyright © 2021 Madill et al. 2021 Madill et al. https://creativecommons.org/licenses/by/4.0/ This content is distributed under the terms of the Creative Commons Attribution 4.0 International license.

10.1128/mSystems.00712-21.5 FIG S7 Phylogenetic analysis of partial 16S rRNA gene sequences of the *Nitrospira* ASVs detected in this study (ASV_8, ASV_32, and ASV_47) (shown in red) in comparison to reference sequences of related *Nitrospira* species obtained from the NCBI. To construct the phylogenetic tree, barrnap v0.9 (https://github.com/tseemann/barrnap) was first used to extract the 16S rRNA gene sequences from the full or partial NCBI reference genomes. The extracted reference 16S rRNA genes were then aligned to the partial 16S rRNA sequences of the three *Nitrospira* ASVs obtained in this study using MUSCLE (R. C. Edgar, Nucleic Acids Res 32:1792–1797, 2004, https://doi.org/10.1093/nar/gkh340), and the multiple-sequence alignment was trimmed in MEGAX (S. Kumar, G. Stecher, M. Li, C. Knyaz, and K. Tamura, Mol Biol Evol 35:1547–1549, 2018, https://doi.org/10.1093/molbev/msy096). The phylogenetic tree was constructed using iq-tree (L.-T. Nguyen, H. A. Schmidt, A. von Haeseler, and B. Q. Minh, Mol Biol Evol 32:268–274, 2015, https://doi.org/10.1093/molbev/msu300) with default parameters and 1,000 nonparametric bootstrap replicates and then visualized using iTOL (I. Letunic and P. Bork, Nucleic Acids Res, 22 April 2021, https://doi.org/10.1093/nar/gkab301). Branch node numbers represent bootstrap support values of >50%. GenBank accession numbers for the reference *Nitrospira* species are provided in parentheses. Download FIG S7, PDF file, 0.3 MB. Copyright © 2021 Madill et al. 2021 Madill et al. https://creativecommons.org/licenses/by/4.0/ This content is distributed under the terms of the Creative Commons Attribution 4.0 International license.

10.1128/mSystems.00712-21.9 FIG S12 Normalized read counts of two predominant *Nitrosomonas* ASVs in triplicate BONCAT-FACS (i.e., BONCAT^+^) and corresponding post-homogenized (Post-Hom.) libraries generated from samples prepared from nitrifying microcosms seeded with mixed liquor preceding return sludge treatment (R), return sludge after 15 min of sidestream treatment (S1), and return sludge after 24 h of sidestream treatment (S2) from the treatment SBR (bottom) and the control SBR (top). Points indicate triplicate normalized read counts per ASV, and the horizontal bars of the same colors represent the sample mean per ASV. The reads were normalized with DESeq2 v.1.24.0 based on total read counts per sample. A black bracket between an ASV within two samples represents a significant difference in the mean read abundance, determined with DESeq2 at an adjusted significance level of a *P* value of <0.01. These were the only two *Nitrosomonas* ASVs that showed differential abundances between BONCAT^+^ and corresponding post-homogenized samples. Download FIG S12, PDF file, 0.2 MB. Copyright © 2021 Madill et al. 2021 Madill et al. https://creativecommons.org/licenses/by/4.0/ This content is distributed under the terms of the Creative Commons Attribution 4.0 International license.

### Substrate analog probing of active nitrifying populations.

**FIG 5:**
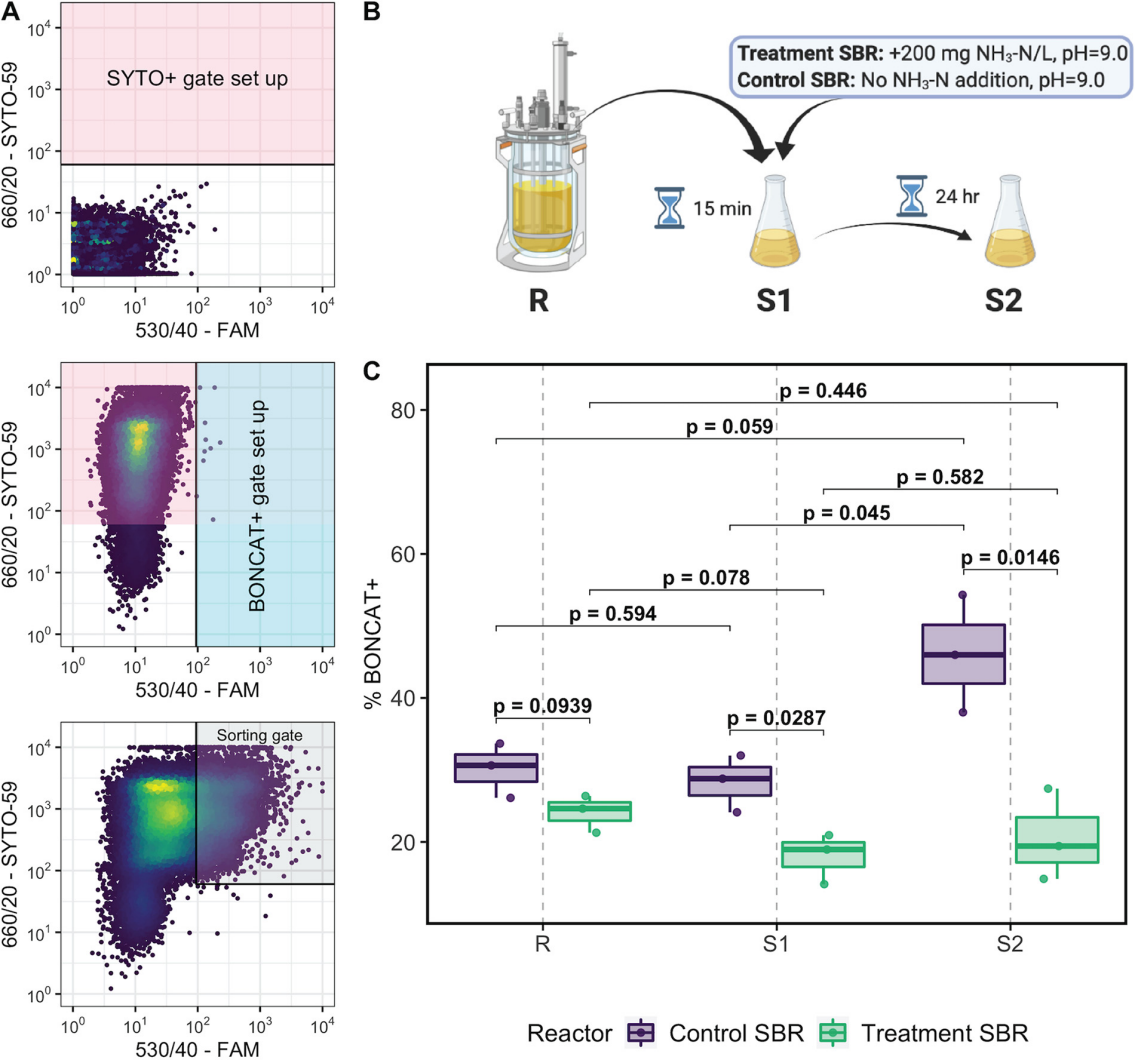
Application of BONCAT-FACS to aerobic nitrifying microcosm samples. (A, top and middle) Sorting gates were established based on the detection of SYTO^+^ cells from background particles using unstained HPG-negative cells (top) and BONCAT^+^ cells from non-HPG-labeled cells based on their FAM picolyl azide dye fluorescence using SYTO59-stained HPG-negative cells (middle), allowing a false-positive rate of <0.5% in each gate. (Bottom) BONCAT^+^ cell fractions in each HPG-incubated sample were calculated as the fraction of SYTO^+^ cells residing in the sorting gate. (B) Overview of the return sludge treatment processes showing the origin of samples used to seed the nitrifying microcosms. (C) Comparison of BONCAT^+^ cell fractions detected in samples prepared from the nitrifying microcosms seeded with mixed liquor preceding return sludge treatment (R), return sludge after 15 min of sidestream treatment (S1), and return sludge after 24 h of sidestream treatment (S2) from the treatment SBR (bottom) and control SBR (top). Black brackets are shown for comparisons of BONCAT^+^ cell fractions made between samples, with *P* values calculated using independent *t* tests.

To decipher the impact of the applied press disturbance on the activity and *in situ* physiology of nitrifying populations within mainstream AS, we conducted BONCAT-FACS on samples collected from nitrifying microcosms seeded with SBR mixed liquor preceding return sludge treatment (R), as well as return sludge from the beginning (15 min after the start [S1]) and end (24 h after the start [S2]) of the sidestream treatment process, for each SBR (Fig. 5). Preliminary validation microcosms confirmed the sensitivity of BONCAT in labeling only active cells that had incorporated HPG (Fig. S8). Comparison of FACS data between the two SBRs (Fig. S9 and S10) revealed that translationally active (i.e., BONCAT-positive [BONCAT^+^]) cell fractions were significantly lower in the S1 and S2 nitrifying microcosms seeded from the FA-exposed treatment SBR than those of the control SBR (*P < *0.05 [*t* test]), but that no significant difference in BONCAT^+^ cell fractions was observed in the R microcosms seeded with mixed liquor preceding sludge treatment (Fig. 5). Within the treatment SBR nitrifying microcosms, the fraction of BONCAT^+^ cells in the S1 microcosm was 25% lower than that in the R microcosm without FA exposure, but this was not significant (*P *= 0.078) (Fig. 5). No further reduction in the fraction of BONCAT^+^ cells was observed between microcosms seeded with return sludge from the beginning (S1) and end (S2) of the FA exposure process (Fig. 5). In contrast, the BONCAT^+^ cell fraction in the microcosm seeded with control SBR return sludge after 24 h of its sludge treatment (S2) was significantly higher (by 63%) than that at the beginning of its sludge treatment process (S1) (*P* = 0.045) (Fig. 5).

10.1128/mSystems.00712-21.7 FIG S9 BONCAT-FACS results generated from treatment SBR microcosm samples. BONCAT-FACS was conducted on post-homogenized samples prepared from nitrifying microcosms seeded with the treatment SBR mixed liquor preceding return sludge treatment (R) (A), return sludge after 15 min of sidestream treatment (S1) (B), and return sludge after 24 h of sidestream treatment (S2) (C). BONCAT^+^ cells residing in the predetermined sorting gate were sorted and collected from each sample for 16S rRNA gene amplicon sequencing. The fractional abundance of BONCAT^+^ cells in each sample was calculated as the fraction of SYTO^+^ cells residing in the sorting gate, as indicated in red text in each box. Download FIG S9, PDF file, 1.3 MB. Copyright © 2021 Madill et al. 2021 Madill et al. https://creativecommons.org/licenses/by/4.0/ This content is distributed under the terms of the Creative Commons Attribution 4.0 International license.

10.1128/mSystems.00712-21.8 FIG S10 BONCAT-FACS results generated from control SBR microcosm samples. BONCAT-FACS was conducted on post-homogenized samples prepared from nitrifying microcosms seeded with the control SBR mixed liquor preceding return sludge treatment (R) (A), return sludge after 15 min of sidestream treatment (S1) (B), and return sludge after 24 h of sidestream treatment (S2) (C). BONCAT^+^ cells residing in the predetermined sorting gate were sorted and collected from each sample for 16S rRNA gene amplicon sequencing. The fractional abundance of BONCAT^+^ cells in each sample was calculated as the fraction of SYTO^+^ cells residing in the sorting gate, as indicated in red text in each box. Download FIG S10, PDF file, 1.2 MB. Copyright © 2021 Madill et al. 2021 Madill et al. https://creativecommons.org/licenses/by/4.0/ This content is distributed under the terms of the Creative Commons Attribution 4.0 International license.

**FIG 6:**
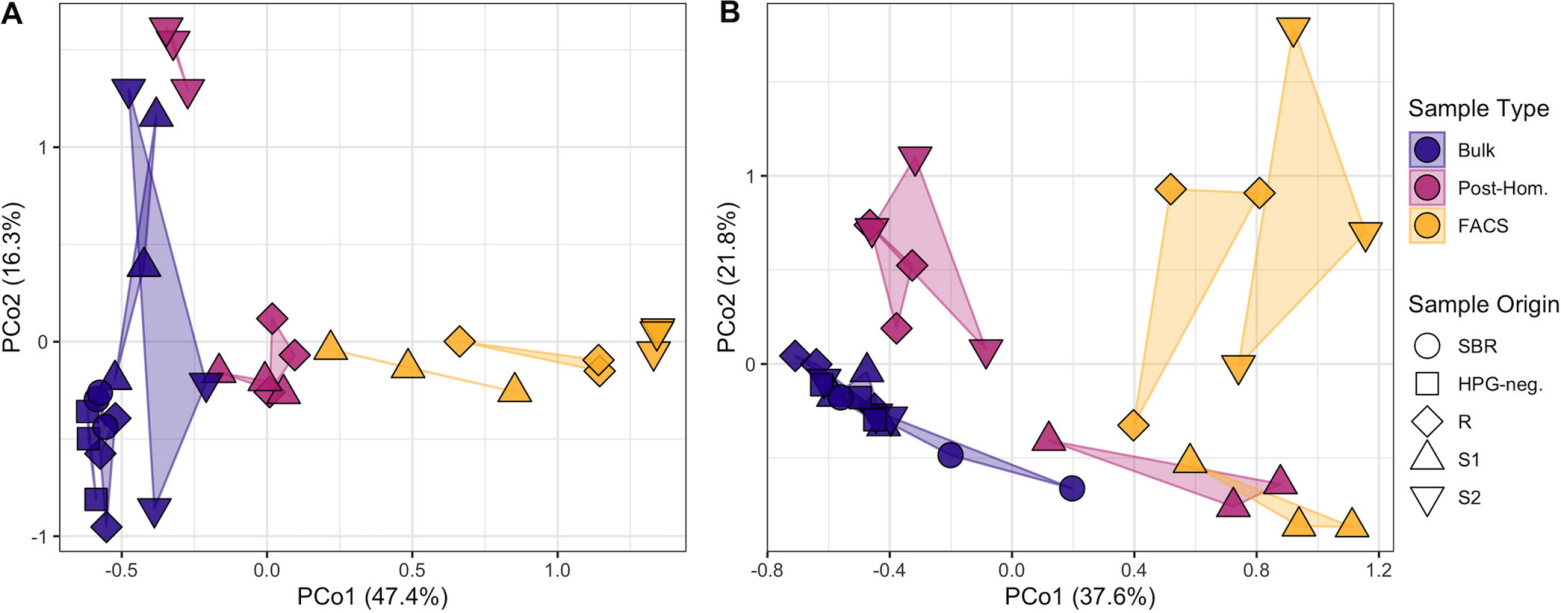
Principal coordinate analysis (PCoA) of Bray-Curtis dissimilarities between cumulative sum-scaled (CSS) read counts of 16S rRNA ASVs measured in bulk, post-homogenized (Post-Hom.), and BONCAT^+^ (i.e., FACS) samples prepared from nitrifying microcosms seeded with mixed liquor preceding sidestream treatment (R), mixed liquor after 15 min of sidestream treatment (S1), and mixed liquor after 24 h of sidestream treatment (S2), from the treatment SBR (A) and the control SBR (B). Bulk samples from the HPG-negative R control microcosms (HPG-neg.) and bulk mixed-liquor samples directly from the SBRs (i.e., SBR) were also included. The marker fill represents the preparation procedure for each sample, and the marker shape represents the sample origin (microcosm type or SBR mixed liquor). Triplicate samples are indicated by a shared polygon. The percentages in parentheses represent the fraction of the variance explained by that coordinate axis.

The microbial community composition observed in the BONCAT^+^ cell fractions could be attributed to changes in cellular translational activity as well as any changes that occurred in the bulk community composition throughout the incubation and/or sample processing steps upstream of FACS. To delineate these impacts, we conducted 16S rRNA gene amplicon sequencing on triplicate click-chemistry-labeled bulk (i.e., pre-homogenized) and post-homogenized samples from each microcosm, in addition to pre-homogenized bulk samples from HPG-negative microcosms and SBR mixed liquors prepared with different DNA extraction and PCR amplification procedures. The low-biomass DNA extraction method (prepGEM kit) and two-step 16S rRNA gene PCR amplification, both of which were used to prepare the pre-homogenized, post-homogenized, and BONCAT^+^ sample libraries, showed impacts on the community composition relative to the respective samples prepared for time series analysis (i.e., FastDNA soil kit with one-step PCR) (see Fig. S11 at https://doi.org/10.6084/m9.figshare.14787984). For this reason, the community compositions measured in the BONCAT^+^ samples were not compared to those of time series samples prepared with different DNA extraction and amplification procedures. PCoA revealed that bulk samples from microcosms incubated without HPG were similar to those of microcosms incubated with HPG, indicating that HPG did not alter the community structure during the 3-h incubation (Fig. 6). Bulk samples from the control SBR microcosms (R, S1, and S2) generally clustered with the mixed liquor sampled directly from the SBR (Fig. 6B). In contrast, bulk samples from the treatment SBR microcosms diverged slightly from the bulk SBR community after 24 h of FA exposure (e.g., S2 microcosms) (Fig. 6A), suggesting that the community composition was altered by exposure to FA. The community compositions of the post-homogenized samples were not identical to those of the bulk microcosm samples, which could be attributed to cell lysis or the removal of extracellular DNA during the homogenization and click-labeling procedures (45) (Fig. 6). Due to the above-mentioned findings, the BONCAT^+^ cell fractions were compared only to their corresponding post-homogenized microcosm samples.

**FIG 7:**
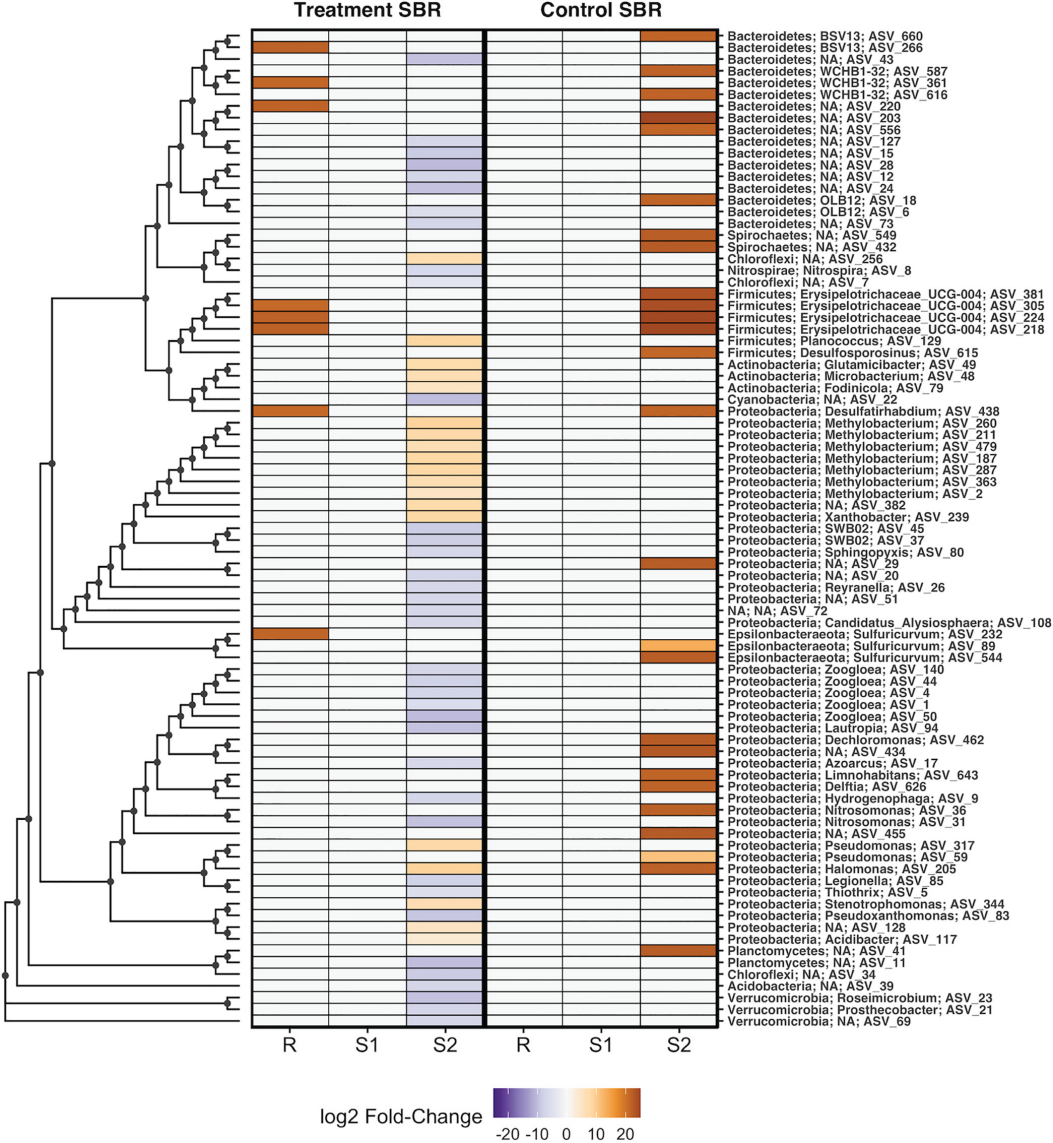
Log_2_ fold changes in abundances of all differentially abundant ASVs detected in comparisons between BONCAT^+^ and post-homogenized fractions prepared from nitrifying microcosms seeded with mixed liquor preceding return sludge treatment (R), return sludge after 15 min of sidestream treatment (S1), and return sludge after 24 h of sidestream treatment (S2) from the treatment SBR (left) and the control SBR (right). For each microcosm, log_2_ fold changes are shown only for differentially abundant ASVs, as determined by comparing ASV abundances in triplicate DNA extracts of each fraction using DESeq2 with an adjusted significance level of a *P* value of <0.01. Log_2_ fold changes that were not significant are set to zero for visualization purposes. ASVs are ordered based on their phylogenetic distances estimated through multiple-sequence alignment using the DECIPHER package (v.2.14.0) (77), and the phylogenetic tree was constructed using a maximum likelihood approach in the phangorn package (v.2.5.5) (78) in the R environment. Tree tip labels (right side of the heat map) denote the phylum- and genus-level classifications of each ASV, where NA denotes an unknown taxonomic identity at that level.

Comparing the abundance of taxa in a BONCAT^+^ cell fraction to that in its corresponding bulk community prior to FACS can identify changes in translational activity at the population level (45, 48). For both the treatment and control SBRs, PCoA showed that the largest distance between post-homogenized community compositions and BONCAT^+^ cell fractions was observed in microcosms seeded with S2 biomass after 24 h of return sludge treatment (Fig. 6). Differential abundance analysis identified 8 and 0 differentially abundant ASVs (*P < *0.01 [DESeq2]) in the BONCAT^+^ fractions of the treatment and control SBR mixed-liquor-seeded (R) microcosms, respectively, and 0 differentially abundant ASVs in the BONCAT^+^ fractions of both SBR microcosms seeded with S1 biomass from the start of return sludge treatment. Conversely, for microcosms seeded with S2 biomass after 24 h of return sludge treatment, 56 and 26 ASVs were differentially abundant in the BONCAT^+^ fractions of the treatment SBR and control SBR samples, respectively. Within the S2 microcosm BONCAT^+^ fraction from the treatment SBR, the 56 differentially abundant ASVs spanned 26 genera, where 34% of the ASVs were significantly enriched and 66% were significantly reduced relative to the post-homogenized community (Fig. 7). In contrast, the 26 differentially abundant ASVs identified in the S2 microcosm BONCAT^+^ fraction from the control SBR, which spanned 13 genera, were all (100%) enriched relative to the post-homogenized community (Fig. 7). These results suggest that sidestream return sludge treatment caused distinct shifts in the translationally active fraction of the communities in both SBRs, with FA exposure negatively impacting the activity of a greater number of taxa than a similar incubation without FA.

**FIG 8:**
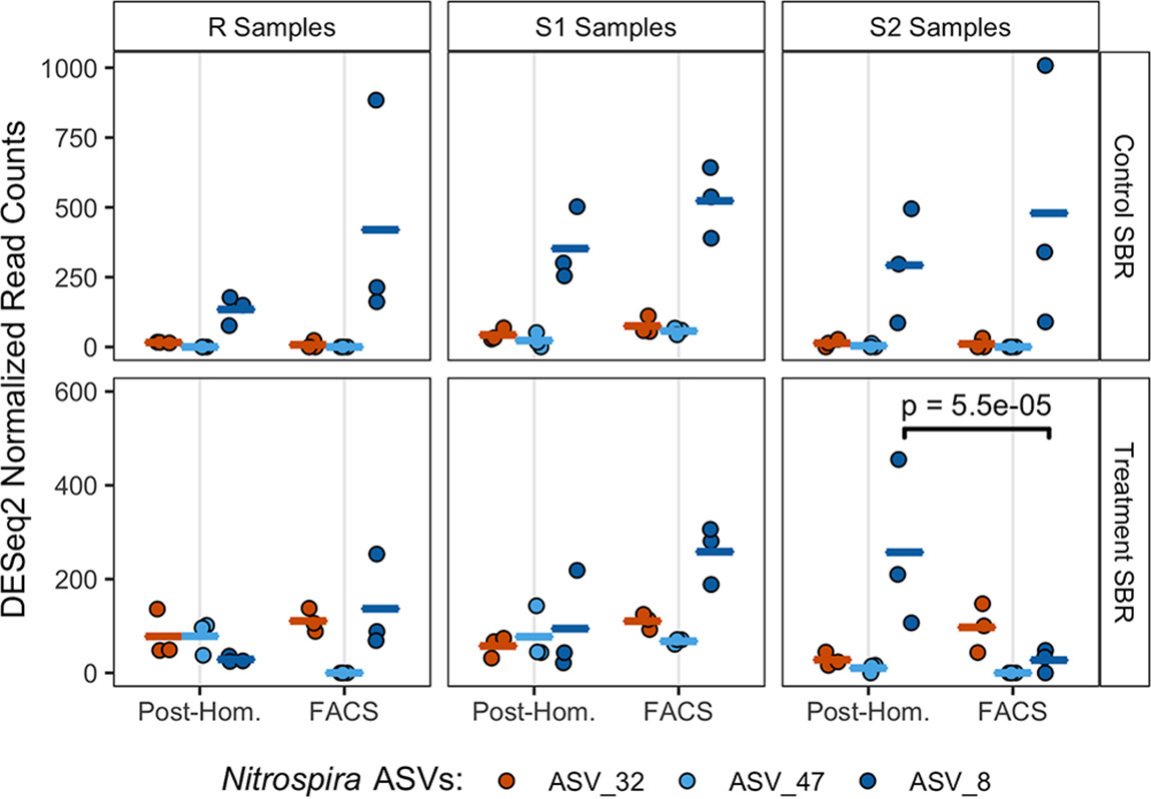
Normalized read counts of three dominant *Nitrospira* ASVs in triplicate BONCAT^+^ (i.e., FACS) and corresponding post-homogenized (Post-Hom.) libraries generated from samples prepared from nitrifying microcosms seeded with mixed liquor preceding return sludge treatment (R), return sludge after 15 min of sidestream treatment (S1), and return sludge after 24 h of sidestream treatment (S2) from the treatment SBR (bottom) and the control SBR (top). Points indicate triplicate normalized read counts per ASV, and the horizontal bars of the same colors represent the sample means per ASV. The reads were normalized with DESeq2 based on total read counts per sample. A black bracket between an ASV within two samples represents a significant difference in the mean read abundance, determined with DESeq2 at an adjusted significance level of a *P* value of <0.01.

As the BONCAT microcosms were amended with NH_4_^+^-N as the sole electron donor, it was possible to assess the impact of return sludge treatment on the translational activity of nitrifying populations. The three dominant *Nitrospira* ASVs and the top two dominant *Nitrosomonas* ASVs detected in both SBR BONCAT microcosm sample sets corresponded to the same dominant ASVs detected within both SBR mixed liquors on day 94 of the time series sampling, when the microcosms were established (Fig. 8; Fig. S12). The two dominant *Nitrosomonas* ASVs (ASV_31 and ASV_36) were both differentially abundant in the BONCAT^+^ fractions of S2 microcosms (*P < *0.01 [DESeq2]) (Fig. S12), while the observed differences varied by SBR. *Nitrosomonas* ASV_31 was significantly reduced in the BONCAT^+^ fraction of the S2 microcosm from the treatment SBR, while *Nitrosomonas* ASV_36 was significantly enriched in the BONCAT^+^ fraction of the S2 microcosm of the control SBR (Fig. S12). Similar to *Nitrosomonas*, the only significant differential abundance in BONCAT^+^ fractions for *Nitrospira* ASVs occurred in an S2 microcosm (Fig. 8). *Nitrospira* ASV_8 was the only differentially abundant NOB in BONCAT^+^ fractions of both SBR microcosms and was significantly reduced in the S2 microcosm of the treatment SBR (*P < *0.01 [DESeq2]). These results indicate that significant reductions in the translational activity of nitrifying populations were observed only in return sludge exposed to FA.

## DISCUSSION

Implementing process control strategies that achieve consistent modulation of key functional groups remains a critical challenge toward the development of sustainable wastewater treatment biotechnologies. This is particularly true for repressing functionally diverse NOB to promote nitritation for energy-efficient biological nitrogen removal in AS processes. This study demonstrates how underlying shifts in the abundance of physiologically diverse *Nitrospira* populations can confer resilience to the nitrite-oxidizing community during an imposed press disturbance in AS treatment, in this case induced by routine exposure of return sludge to FA. Our results also demonstrate the utility of substrate analog probing approaches like BONCAT to illuminate the *in situ* ecophysiology of shared niches within the activated sludge microbiome and the associated impacts on process and ecosystem functional stability.

We observed that routinely exposing ∼20% of the return sludge to FA (200 mg NH_3_-N/liter) in a sidestream reactor with a 24-h retention time initially reduced the nitrite-oxidizing activity in the mainstream reactor, achieving a maximum observed NAR of 42%. However, we observed acclimation of the nitrite oxidation function after approximately 40 days of the press disturbance, indicated by decreasing NARs within the treatment SBR. This finding is in contrast to those reported by Wang et al. (21), who observed stable repression of NOB activity for over 100 days using a press disturbance of routine FA exposure under similar conditions (210 mg N/liter of FA for 24 h; 22% of return sludge exposed). It is important to note that both the pH of the sidestream sludge treatment incubation and the daily input of total ammonia nitrogen into the mainstream SBR with the return sludge were controlled across both SBRs. Furthermore, while salinity has been shown to be a driver of *Nitrospira* population structure (53), the salinity in the sidestream reactor during FA exposure (∼2.5 g/liter as Na^+^ plus Cl^−^) was 10 times lower than the 50% inhibition level observed for a *Nitrospira*-dominated AS community of 30 g/liter NaCl (54). Therefore, the inhibition and acclimation of NOB activity to the applied press disturbance can likely be attributed to various physiological responses of NOB community members to FA exposure.

While acclimation of NOB communities in mainstream AS to routine FA exposure has been previously reported (41, 42, 55), very few studies have directly investigated the role of physiological diversity between NOB members in supporting community-level acclimation. To our knowledge, all previous reports of NOB community acclimation to return sludge FA exposure have involved shifts in major NOB genera during this press disturbance. Specifically, Li et al. (42) found that *Candidatus* Nitrotoga replaced *Nitrospira* as the dominant NOB in response to return sludge FA exposure, while Duan et al. (41) reported a shift from *Nitrospira* to *Nitrobacter* in response to FA exposure. These reported shifts could be supported by potentially distinct physiological characteristics of *Candidatus* Nitrotoga and *Nitrobacter* compared to *Nitrospira*, such as higher tolerances to FA inhibition (42, 56) and/or preferences for higher nitrite concentrations (30, 57). In contrast to these genus-level community shifts, however, we observed that NOB community acclimation to FA exposure could occur via shifts between *Nitrospira* sequence variants, specifically from a dominant variant belonging to *Nitrospira* lineage II (ASV_8) to two variants that belonged to *Nitrospira* lineage I (ASV_32 and ASV_47). These findings therefore reveal that physiological diversity at the lineage level, and possibly at the sublineage or strain level, within *Nitrospira*-dominated communities can facilitate niche partitioning and acclimation to FA exposure as a press disturbance when applied as an engineering control strategy to promote energy-efficient nitrogen removal.

*Nitrospira* species are well known to be metabolically versatile, with a wide range of functional potentials, including nitrite oxidation, hydrogen oxidation, urea conversion, formate oxidation, nitrate reduction, and complete ammonium oxidation (24, 37, 38, 52, 58). This metabolic diversity likely creates opportunities for functionally degenerate *Nitrospira* populations to coexist within a bioreactor by differentiating in niche space through auxiliary metabolic specializations while also sharing a niche space of nitrite oxidation (34, 59). Here, we employed BONCAT-FACS for the first time in an AS wastewater treatment system, to the best of our knowledge, which highlighted differing *in situ* physiologies of three *Nitrospira* variants and resolved their responses to FA exposure on the level of cellular translational activity. BONCAT-FACS revealed that transitional activity in *Nitrospira* ASV_8 was significantly reduced following exposure to FA for 24 h, aligning with the results of the time series reactor sampling in which *Nitrospira* ASV_8 was washed out of the treatment SBR but remained dominant in the control SBR. In contrast, *Nitrospira* ASV_32 and ASV_47 increased in abundance within the treatment SBR, coinciding with the decrease in the NAR after day 40, and BONCAT-FACS revealed that the translational activity of these variants remained unchanged following FA exposure. Based on the close alignment of the BONCAT-FACS observations with the trends in the time series reactor sampling, it can therefore be hypothesized that the washout of *Nitrospira* ASV_8 occurred due to its decreased activity in response to the press disturbance of routine sidestream FA exposure, which induced a growth lag once it was reintroduced into the mainstream SBR. In contrast, the potentially distinct auxiliary metabolic potentials of *Nitrospira* ASV_32 and ASV_47 may have conferred their physiological resistance to the sidestream FA exposure, thereby providing these variants with a competitive growth advantage within the mainstream SBR. Physiological tolerance to FA exposure was identified as a mechanism of niche partitioning between *Nitrospira* populations of lineages I and II by Ushiki et al. (40), who showed that *Nitrospira* sp. strain ND1 of lineage I was more sensitive to FA than Nitrospira japonica of lineage II at 100 mg NH_4_^+^-N/liter at pH 8.0. Here, we observed that *Nitrospira* ASV_32 and ASV_47 of lineage I were physiologically more tolerant to FA than *Nitrospira* ASV_8 of lineage II, suggesting that either lineage-specific tolerances are distinct at the higher FA concentration applied here (200 mg NH_3_-N/liter) or physiological tolerance to FA is a trait that varies at the strain/species level within *Nitrospira*. Regardless, once *Nitrospira* ASV_32 and ASV_47 grew to high-enough abundances in the treatment SBR, they likely contributed to the net oxidation of nitrite and the decrease in the NAR that was observed after day 40 of the treatment phase. Therefore, the FA exposure press disturbance strategy employed in this study acted as a selective pressure that impacted the stability of the NOB community composition, and functional degeneracy within the *Nitrospira* sequence variants likely provided the nitrite-oxidizing community with resilience by shifting activity to physiologically resistant community members.

The above-described results highlight the need to combine press disturbances that target the distinct physiological traits of multiple functionally degenerate NOB populations, defined at both the inter- and intragenus level, to effectively reduce the aggregate activity of the NOB community for energy-efficient nitrogen removal. For example, if future studies support our finding that lineage I *Nitrospira* populations are more tolerant to FA than those of lineage II, then combining press disturbances that further target the physiological traits of lineage I *Nitrospira* along with FA exposure could prove efficacious. Such strategies could entail the maintenance of high dissolved oxygen concentrations, based on the adaptation of lineage I *Nitrospira* to low-dissolved-oxygen environments (34, 36), or the maintenance of low nitrite concentrations based on their preference for higher nitrite concentrations (34, 40, 51). Measurements of maximal growth rates for these sequence variants could also inform washout strategies based on limiting the solids retention time (SRT) (13). Acknowledging the potentially tight tolerances and dynamic nature of nitrite, oxygen, and SRT controls required to target the biokinetics of lineage I *Nitrospira* in full-scale AS systems, combinatorial press disturbance strategies would likely benefit from control strategies that incorporate frequent community monitoring and biokinetic data of these physiologically diverse NOB populations. Overall, these findings underscore the need for broader applications of *in situ* physiology approaches for elucidating the impacts of NOB outselection strategies on functionally active NOB members within activated sludge systems.

Accurately measuring the active biomass fraction in microbial bioprocesses is critical, as many key biokinetic models and process mass balances are based on active biomass concentrations (60). However, conventional indirect approaches for quantifying active biomass based on net substrate utilization and growth yields are unable to resolve the compositional and functional dynamics of active microbial populations. As enzymes are the key catalysts that drive the majority of substrate transformations in microbial bioprocesses, we posit that measures of active biomass should be based on the translationally active microbial cell fraction. The translationally active cell fractions in both SBR microcosms seeded with mixed liquor measured by BONCAT-FACS (R) (24.1% ± 2.6% for the treatment and 30.1% ± 3.8% for the control) were smaller than the active biomass fraction predicted through steady-state modeling (61) (67 to 77% VSS basis) (see Text S1 in the supplemental material), which is likely because the microcosms were supplemented with only ammonium as an electron donor. Nonetheless, we observed many translationally active heterotrophs in the BONCAT microcosms, which could have remained active through the metabolism of internal carbon reserves, endogenous respiration, or constitutive protein expression. The consistent increase in translational activity across all differentially abundant ASVs detected in the BONCAT^+^ fraction of the control SBR microcosm seeded with sludge following the 24-h sidestream incubation could thus have been attributed to fermentative metabolism on cell decay products. In contrast, the mixed translational responses of differentially abundant ASVs in the BONCAT^+^ fraction of the treatment SBR microcosm following sidestream incubation with FA suggest that more complex dynamics between cellular inhibition, decay, and fermentative metabolism were induced by FA exposure. This analysis highlights the value of BONCAT as an *in situ* physiology approach to directly measure concentrations of translationally active population members in microbial bioprocesses. This approach could therefore be extended to provide actual measurements of the active biomass concentration for use in calibrating and validating higher-resolution process models, which have been called for as tools to optimize energy-efficient nitrogen removal technologies (9), as well as to better resolve biogeochemical cycles in natural ecosystems (62, 63). BONCAT-FACS could also be extended to resolve associations in the activity of AS taxa with different substrate preferences (48), thus helping to validate ecological-scale models of the wastewater microbiome (64). Therefore, *in situ* physiology approaches like BONCAT show great promise to help inform new strategies to model, control, and engineer microbiome function in environmental biotechnologies and natural ecosystems alike.

## MATERIALS AND METHODS

### Reactor setup, operation, and monitoring.

Two identical laboratory-scale SBRs with working volumes of 4.28 liters were seeded with AS from a pilot-scale SBR at a WWTP in King County, WA (see Fig. S1 in the supplemental material). The SBR cycles lasted 3 h, including 2 min of aerobic feeding, 148 min of aerobic reaction, 20 min of settling, 5 min of decanting, and 5-min idle periods. The SBR cycle timing was controlled with ChronTrol XT timers (ChronTrol Corporation, USA), and mixing was provided by overhead mixers. The reactor temperature was maintained at 20°C ± 1°C using an environmental chamber. The target SRTs were kept at 10 days for both SBRs throughout the study by wasting a determined amount of biomass based on daily values of TSS and VSS measured in the mixed liquor and effluent streams. The SBRs were fed with synthetic wastewater containing ammonium chloride as the nitrogen source and sodium acetate and propionic acid as organic carbon sources, producing 24.5 ± 2 mg NH_4_^+^-N/liter and 100 ± 37 mg/liter of soluble chemical oxygen demand (sCOD), respectively, to reflect a sCOD/NH_4_^+^-N ratio of ∼4.0 typical of North American wastewaters (61). Macro- and microelement components of the synthetic wastewater are detailed in the supplemental material.

Two operational phases were sequentially conducted: the start-up phase and the treatment phase. In the start-up phase, the two SBRs were operated under the same aerobic conditions without FA exposure of return sludge to achieve similar nitrification performances. The SBRs were fed with 1.07 liters of synthetic wastewater in each SBR cycle using peristaltic pumps (LabF1/YZ1515; Shenchen Precision Pump, China), resulting in a hydraulic retention time (HRT) of 12 h. The pH was not controlled but measured within the range of 6.7 to 7.5. Aeration was provided by an air pump, and dissolved oxygen was not controlled but ranged from 3 to 8 mg/liter in a typical SBR cycle for both reactors. The start-up phase lasted 274 days to establish steady-state conditions.

The treatment phase was commenced on day 275, referred to here as “day zero,” and lasted 94 days. The operational conditions in the treatment phase were similar to those in the start-up phase, except for the following differences (Fig. S1). In the treatment phase, 800 ml of mixed liquor was removed from each SBR at the end of the reaction period of a given cycle every 24 h and thickened to 50 ml by centrifugation. The thickened return sludge (50 ml) was incubated in an unstirred 200-ml beaker along with 100 ml of medium with the same composition as that of the synthetic feed but with no chemical oxygen demand (COD). For the experimental reactor, termed the treatment SBR, the return sludge treatment solution contained 1,060 mg/liter NH_4_^+^-N, with the pH adjusted to 9.0 using sodium hydroxide, to produce a final FA concentration of 200 mg NH_3_-N/liter. These FA and total ammonia nitrogen concentrations are within the ranges observed in anaerobic digester centrates, particularly codigesters and those with thermal hydrolysis pretreatment (65, 66). In the other reactor, termed the control SBR, thickened return sludge was incubated in the same medium at a pH of 9.0 but without ammonium addition. After 24 h of incubation, the 150 ml of treated return sludge was recycled back into the respective SBRs at the start of the next cycle. To maintain consistent nitrogen loadings in the two SBRs, 0.406 g of ammonium chloride was added to the control SBR simultaneously with the treated return sludge. Monitoring experiments lasting 24 h were performed approximately every 10 days to measure composite daily NARs, as described in the supplemental material.

During the treatment phase, effluent nutrient samples were collected just before the addition of treated return sludge. Nitrogen compounds (ammonium, nitrite, and nitrate), orthophosphate, and sCOD in effluent samples were monitored three to four times per week. pH and DO were measured at least three times per week. Analytical methods for bioreactor monitoring are described in Text S1.

### Microcosms for BONCAT.

Three 30-ml samples were collected from each SBR throughout the return sludge treatment cycle commenced on day 94, including (i) a mixed-liquor sample immediately preceding bulk mixed-liquor removal for return sludge treatment (R), (ii) a return sludge sample after 15 min of sidestream treatment (S1), and (iii) a return sludge sample after 24 h of sidestream treatment (S2). The treated return sludge samples were volume corrected for the thickening process to attain the same biomass concentrations as those of the mixed-liquor samples. Samples were washed in phosphate-buffered saline (PBS) (1×; filter sterilized) by centrifugation (3,000 rpm for 5 min) and resuspension to remove residual growth substrates. All samples were incubated in a medium consisting of 25% synthetic wastewater in 1× PBS (vol/vol) without COD or yeast extract so that NH_4_^+^-N was the only exogenous electron donor. For BONCAT labeling microcosms, 15-ml portions of each sample were resuspended in 15 ml of incubation medium amended with 1 mM homopropargylglycine (HPG; Click Chemistry Tools, USA) (HPG-positive microcosm), transferred into sterile 125-ml Erlenmeyer flasks, and incubated on an orbital shaker for 3 h at 20°C at 200 rpm. Control microcosms (HPG-negative) for each sample were conducted similarly except without HPG amendment. Following incubations, microcosm samples were washed three times in 1× PBS to remove unincorporated HPG, resuspended in 10% (vol/vol) glycerol in PBS, aliquoted into 1-ml fractions, and stored at −80°C until further processing. Details on preliminary BONCAT validation microcosms are provided in the supplemental material.

### BONCAT sample preparation and click chemistry.

For each microcosm type (R, S1, or S2) for both SBRs, sample preparation and click chemistry were conducted using triplicate HPG-positive and duplicate HPG-negative microcosm samples. Samples were thawed on ice at 4°C, enzymatically homogenized, subjected to filter-immobilized click chemistry labeling with the 6-carboxyfluorescein (FAM) picolyl azide dye (Click Chemistry Tools, USA) closely following the procedure described previously by Couradeau et al. (45), detached from the filter, pre-strained through a 30-μm-mesh filter, and counterstained with SYTO59 (10,000-fold final dilution; Thermo Fisher Scientific, USA) to generate the post-homogenized samples for fluorescence-activated cell sorting (FACS). Details of the sample homogenization and click chemistry labeling procedures are provided in the supplemental material.

### Fluorescence-activated cell sorting.

Fluorescence-activated cell sorting was conducted on a BD FACSJazz cell sorter (BD Biosciences, USA) calibrated to detect the FAM picolyl azide dye (excitation at 490 nm/emission at 510 nm) and the SYTO59 counterstain dye (excitation at 622 nm/emission at 645 nm). An overview of the FACS gating procedures is provided in the supplemental material. Briefly, initial gating was established with side-scatter, forward-scatter, and trigger pulse width to exclude large particles and cell aggregates. Sorting gates were set to target BONCAT-positive (BONCAT^+^) cell fractions based on background SYTO59 and FAM fluorescence, allowing a false-positive rate of <0.5% (Fig. S13; see also Fig. S14 at https://doi.org/10.6084/m9.figshare.14787984). A total of 100,000 cells were analyzed from each sample, where cells within the sorting gate (SYTO^+^ and BONCAT^+^) were sorted into 1.5-ml tubes containing 400 μl of prepGEM wash buffer (ZyGEM, USA), and stored at −80°C until further analysis.

10.1128/mSystems.00712-21.10 FIG S13 BONCAT-FACS gating setup using treatment SBR microcosm samples that were not stained to determine background fluorescence passing a 660/20-nm filter (A) and not incubated with HPG (B) but exposed to click chemistry with FAM picolyl azide dye and counterstained with SYTO59 to determine background fluorescence passing a 530/40-nm filter. The thresholds for the SYTO^+^ and BONCAT^+^ regions were selected so that the mean false-positive rate was below 0.5%. Download FIG S13, PDF file, 0.6 MB. Copyright © 2021 Madill et al. 2021 Madill et al. https://creativecommons.org/licenses/by/4.0/ This content is distributed under the terms of the Creative Commons Attribution 4.0 International license.

### DNA extraction and 16S rRNA gene amplicon sequencing.

For time series analysis of community composition, triplicate 980-μl aliquots of mixed liquor were routinely collected directly from the SBRs at the end of a cycle and flash-frozen at −80°C. Mixed-liquor samples were thawed on ice, and DNA was extracted using the FastDNA spin kit for soil (MP Biomedicals, USA), with minor modifications (67). For each HPG-positive microcosm type (R, S1, or S2) for both SBRs, 50-μl aliquots were collected from the triplicate pre-homogenized and post-homogenized samples during preparation for FACS, added to 400 μl of prepGEM wash buffer, and stored at −80°C. Pre-homogenized samples from HPG-negative R microcosms and bulk mixed-liquor samples were prepared similarly. All pre-homogenized, post-homogenized, and BONCAT-FACS samples were extracted using the prepGEM bacterial kit (ZyGEM) using a low-biomass input procedure overviewed in the supplemental material. DNA concentrations were measured with Qubit dsDNA BR and HS assay kits and a Qubit fluorometer (Thermo Fisher Scientific, USA).

16S rRNA gene fragments from all DNA extracts were amplified using barcoded primers 515F and 926R (68), targeting the V4-V5 hypervariable region of the 16S rRNA gene. All samples extracted with the prepGEM kit underwent an initial round of 15 cycles of PCR with non-barcoded primers 515F and 926R due to the low-biomass input. One triplicate set of FastDNA extracts from day 94 was also pre-amplified to determine the potential biases of that step. Amplified barcoded PCR products were pooled at equimolar concentrations and sequenced on an Illumina MiSeq platform in paired-end 300-bp mode at the UBC Biofactorial Facility.

Amplicon reads were processed and denoised into amplicon sequence variants (ASVs) with DADA2 (49) (v.1.12.1) in the R environment. The script used to generate the ASV data sets is provided in the supplemental material. Denoised sequences were taxonomically classified using the RDB Classifier (69) against the MiDAS 3.0 database (67).

### Statistical analysis.

Comparisons of reactor nutrient data were performed with *t* tests for N-of-1 trials with serial correlation (70). Amplicon sequencing data were visualized using the tidyverse package (71) (v.1.3.0) in the R environment. Principal-coordinate analysis (PCoA) of cumulative sum-scaled (CSS) ASV read counts was performed using the metagenomeSeq (72) (v.1.26.3) and vegan (73) (v.2.5.6) packages in R. Permutational multivariate analysis of variance (PERMANOVA) (adonis) was conducted in vegan with 1,000 permutations to determine significant differences in reactor communities over time. Differential abundance analysis of ASVs between SBRs and in BONCAT microcosm data sets was performed using DESeq2 (74) (v.1.24.0), using parametric fitting, the Wald significance test, and Benjamini-Hochberg correction for *P* values. A log_2_ fold change of an ASV represents the multiplicative effect size for changes in normalized read counts across treatments on a logarithmic scale to base 2. Sequence similarity values between ASVs were calculated using the NCBI Basic Local Alignment Search Tool (75). FACS data were processed using the flowcore package (v.1.52.1) (76) in R.

### Data availability.

The raw read files of 16S rRNA gene amplicons are available via the NCBI Sequence Read Archive under BioProject accession number PRJNA693634.
