# Supplementary material for: Activity-Based Cell Sorting Reveals Resistance of Functionally Degenerate Nitrospira during a Press Disturbance in Nitrifying Activated Sludge
Source: mSystems. 2021 Jul 20;6(4):e00712-21. doi: 10.1128/mSystems.00712-21 (PMC8407113; doi:10.1128/mSystems.00712-21)
Supplement: FIG S1 [file msystems.00712-21-sf001.pdf]

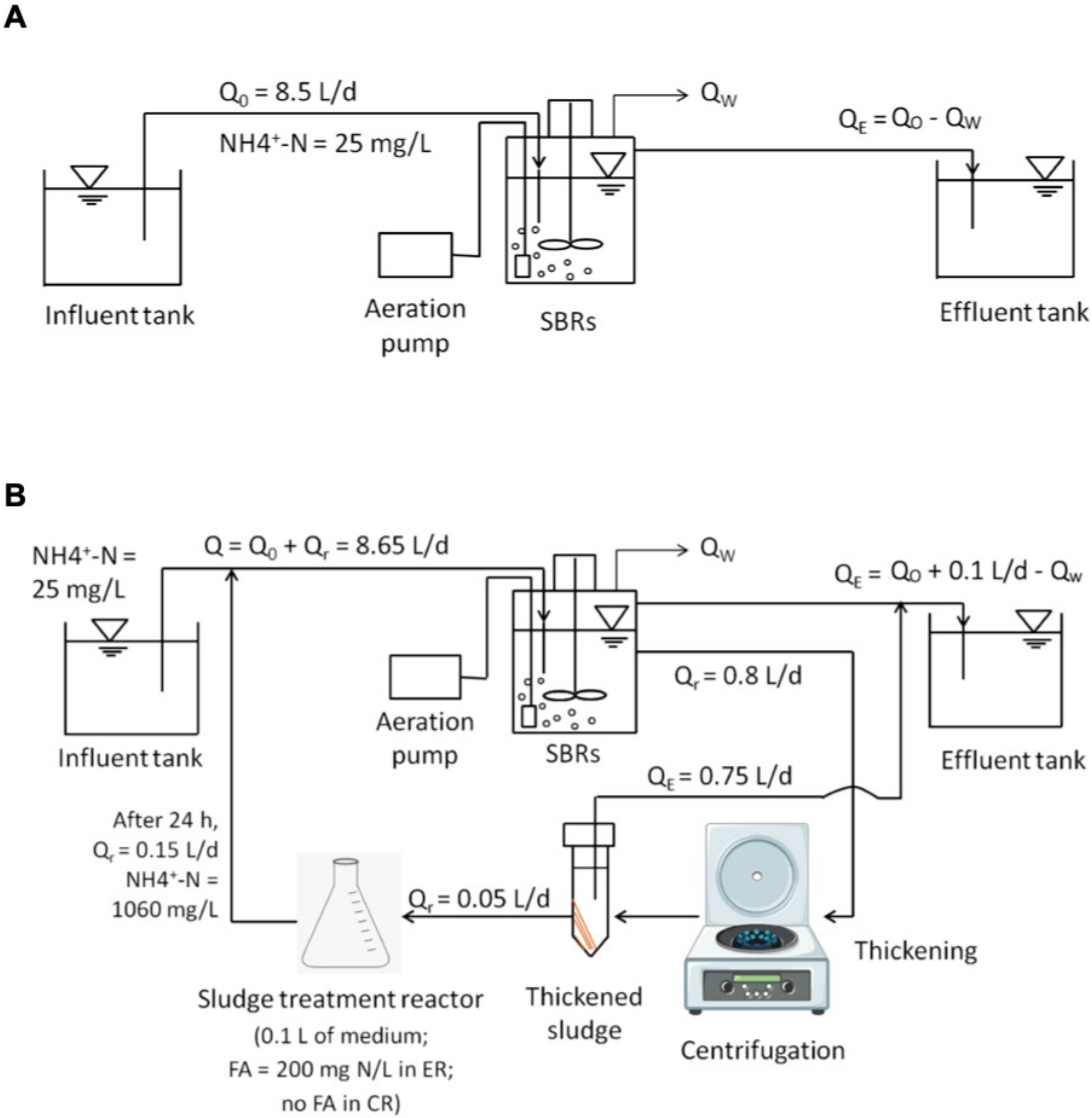
A
Q_0 = 8.5 L/d
$NH4^+$-N = 25 mg/L
Q_W
$Q_E = Q_O - Q_W$
Influent tank
Aeration pump
SBRs
Effluent tank
B
$NH4^+$-N = 25 mg/L
$Q = Q_0 + Q_r$ = 8.65 L/d
Q_W
$Q_E = Q_O$ + 0.1 L/d - Q_w
Influent tank
Aeration pump
SBRs
Q_r = 0.8 L/d
Effluent tank
Q_E = 0.75 L/d
After 24 h, Q_r = 0.15 L/d $NH4^+$-N = 1060 mg/L
Q_r = 0.05 L/d
Thickening
Sludge treatment reactor
(0.1 L of medium;
FA = 200 mg N/L in ER;
no FA in CR)
Thickened sludge
Centrifugation
