# Supplementary figures and images for: Activity-Based Cell Sorting Reveals Resistance of Functionally Degenerate Nitrospira during a Press Disturbance in Nitrifying Activated Sludge
Source: mSystems. 2021 Jul 20;6(4):e00712-21. doi: 10.1128/mSystems.00712-21 (PMC8407113; doi:10.1128/mSystems.00712-21)

**A**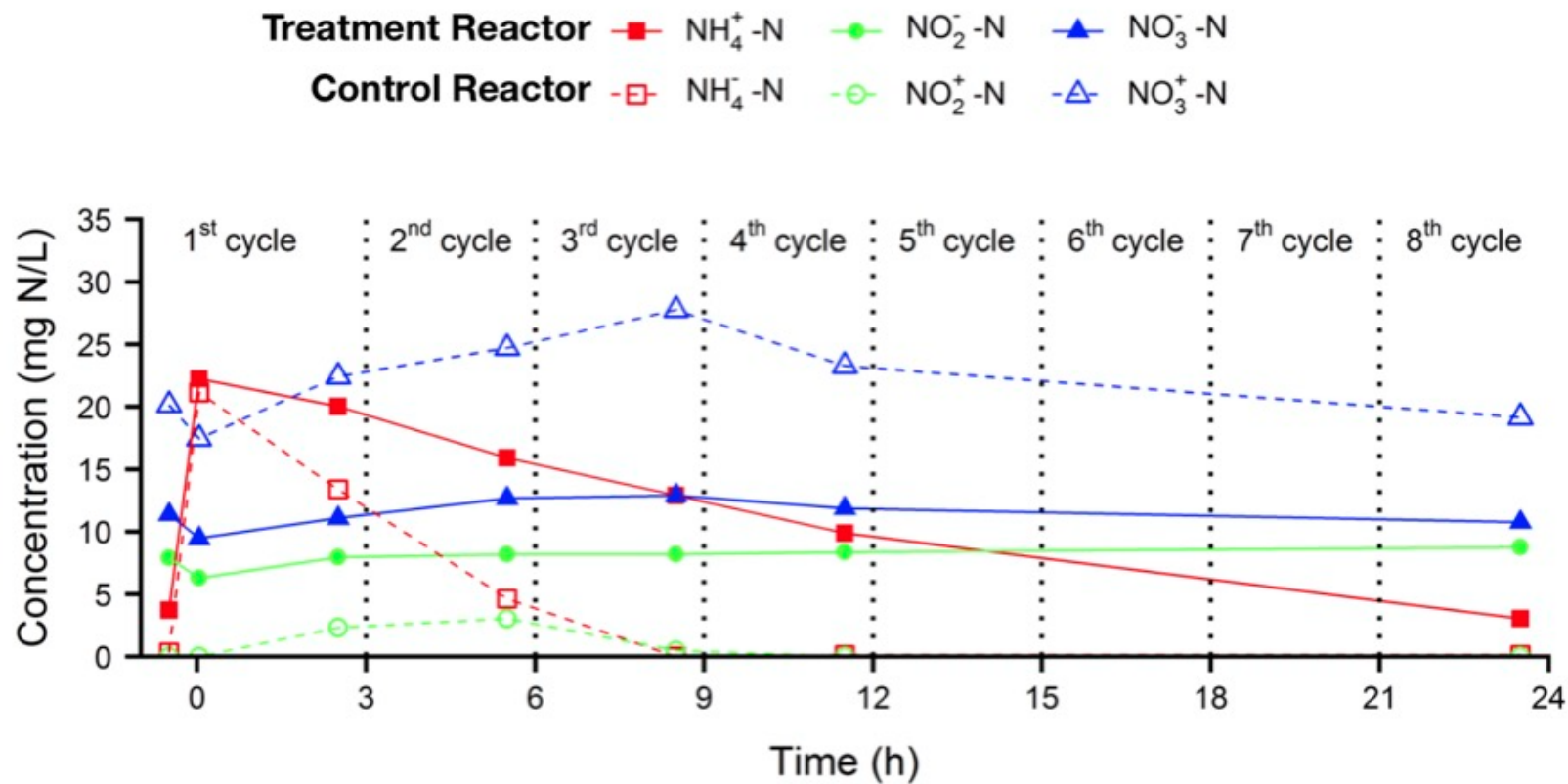**B**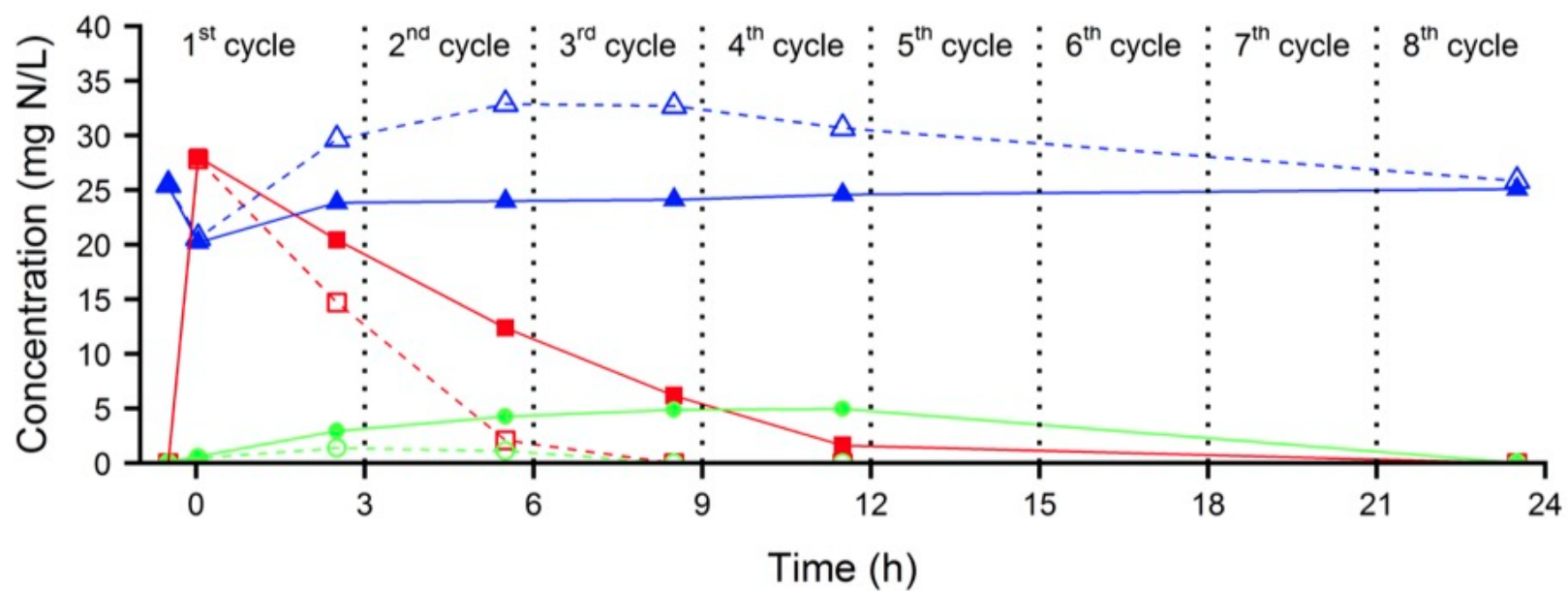

Supplement: FIG S3 [file msystems.00712-21-sf003.pdf]

**A** HPG-Negative Control

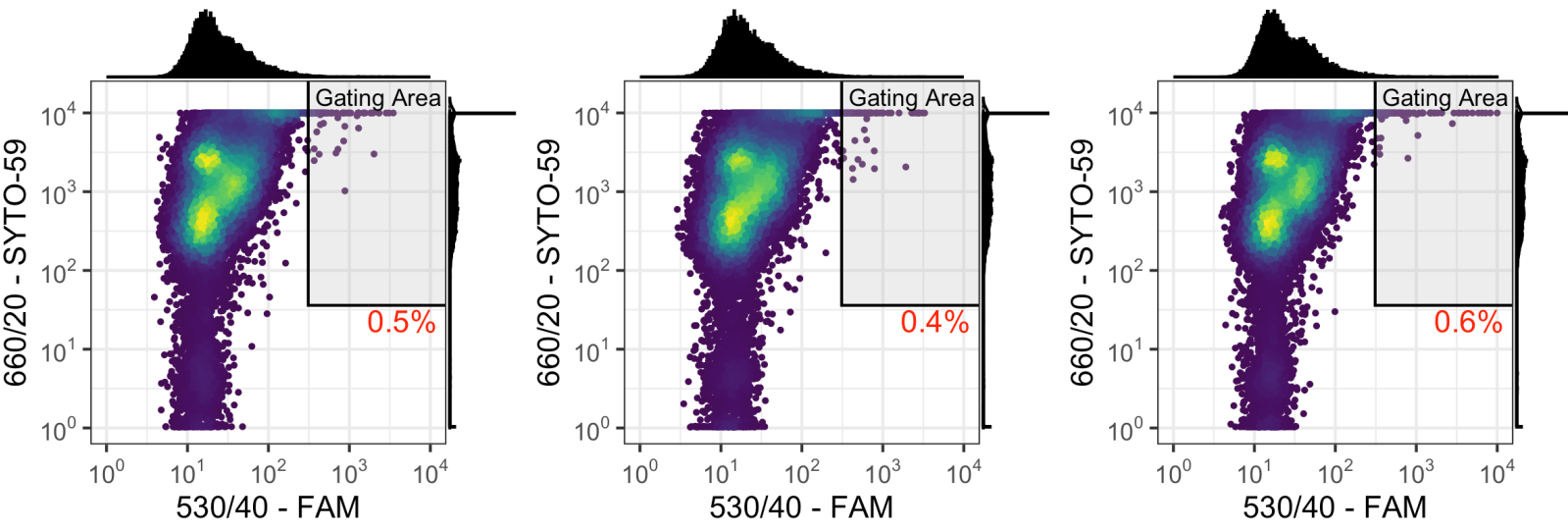

**B** Pre-Incubation Fixed

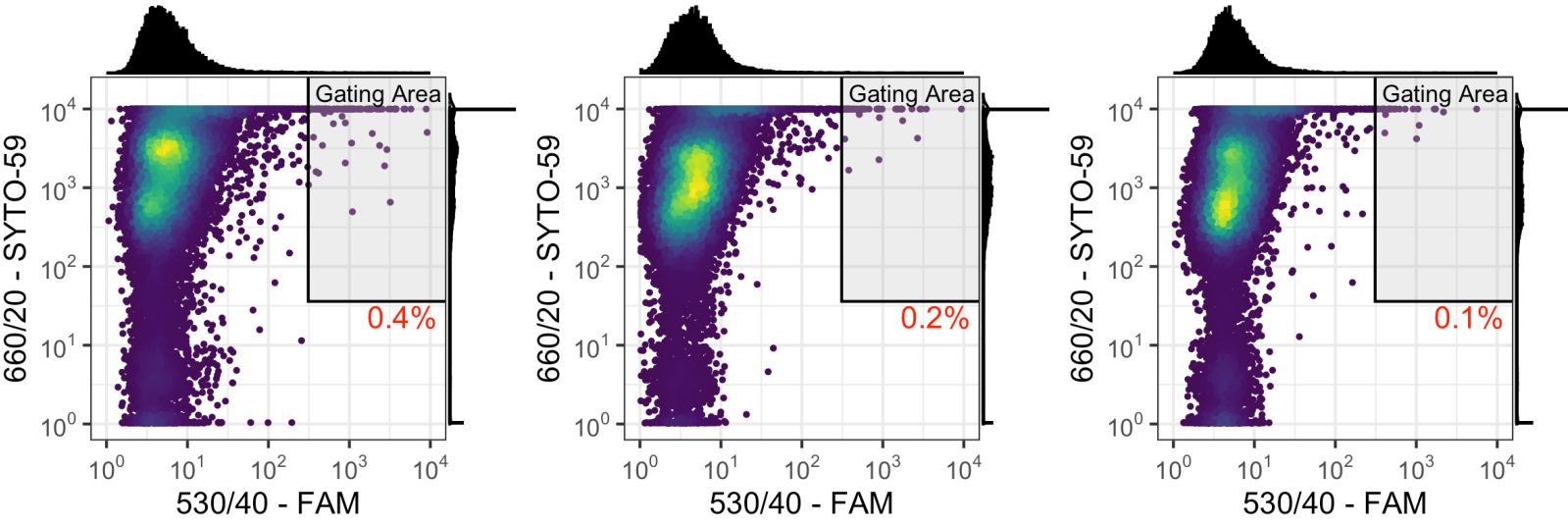

**C** Post-Incubation Fixed

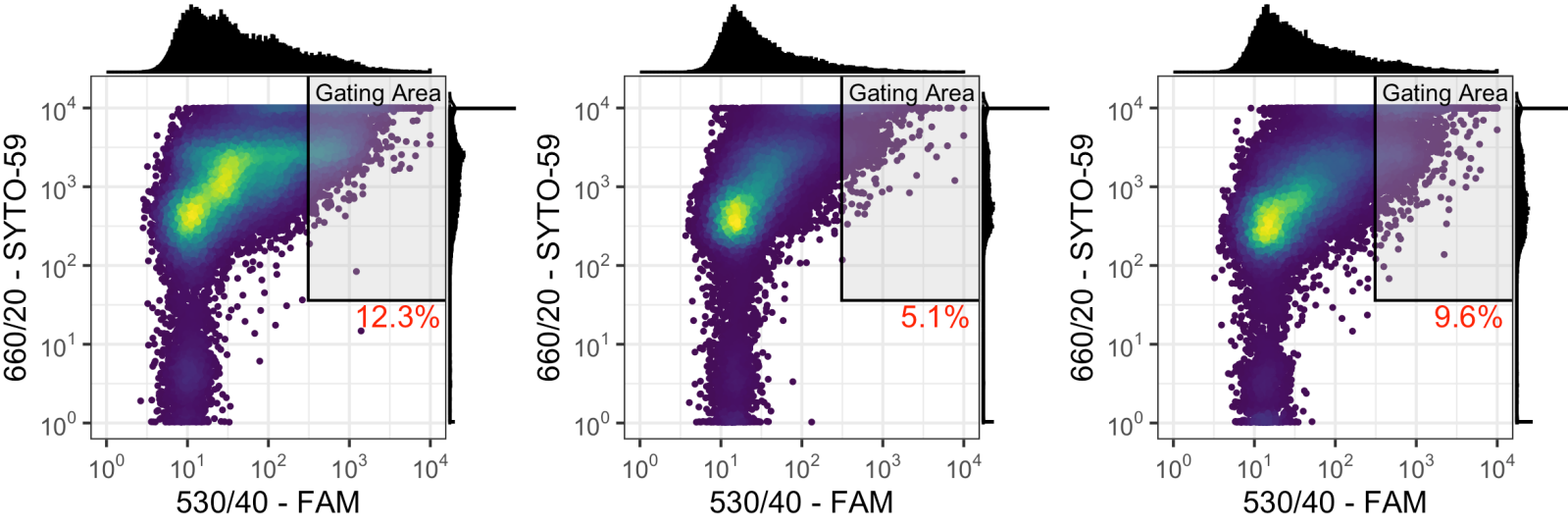

Supplement: FIG S8 [file msystems.00712-21-sf008.pdf]

Relative abundance of 16S rRNA genes (TSS, %)

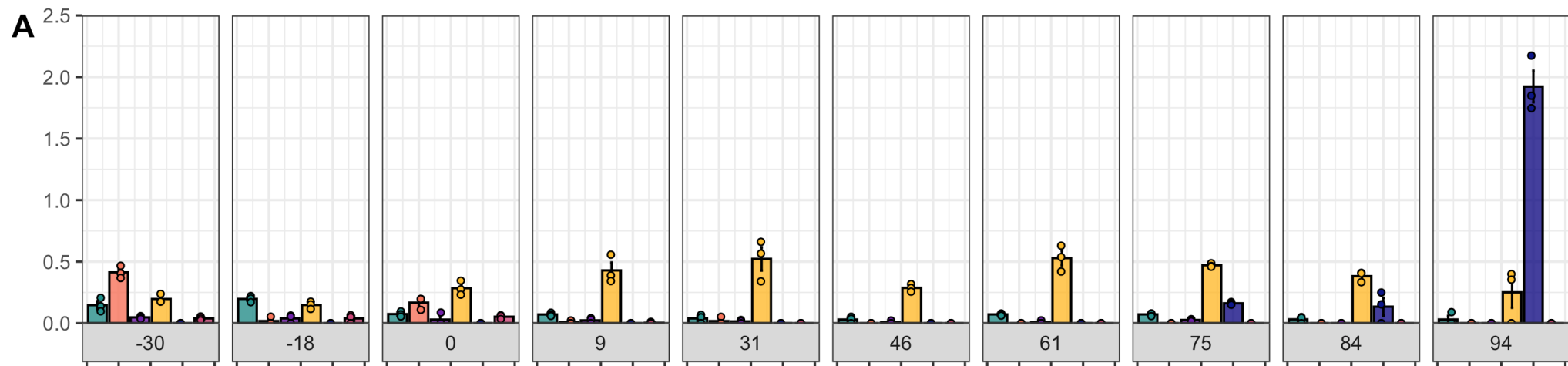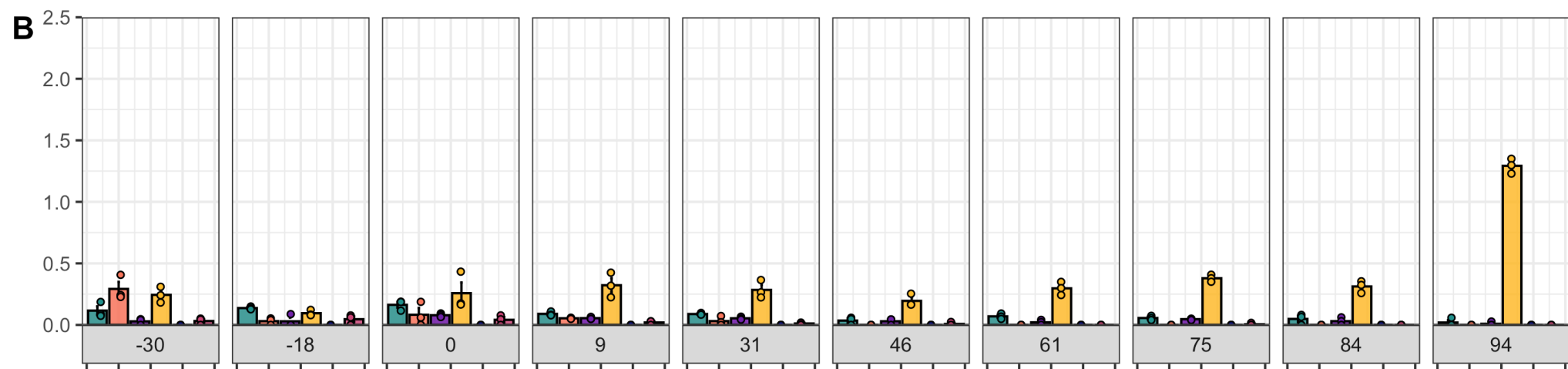

Day

*Nitrosomonas* ASVs:

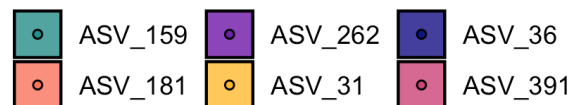

Supplement: FIG S6 [file msystems.00712-21-sf006.pdf]

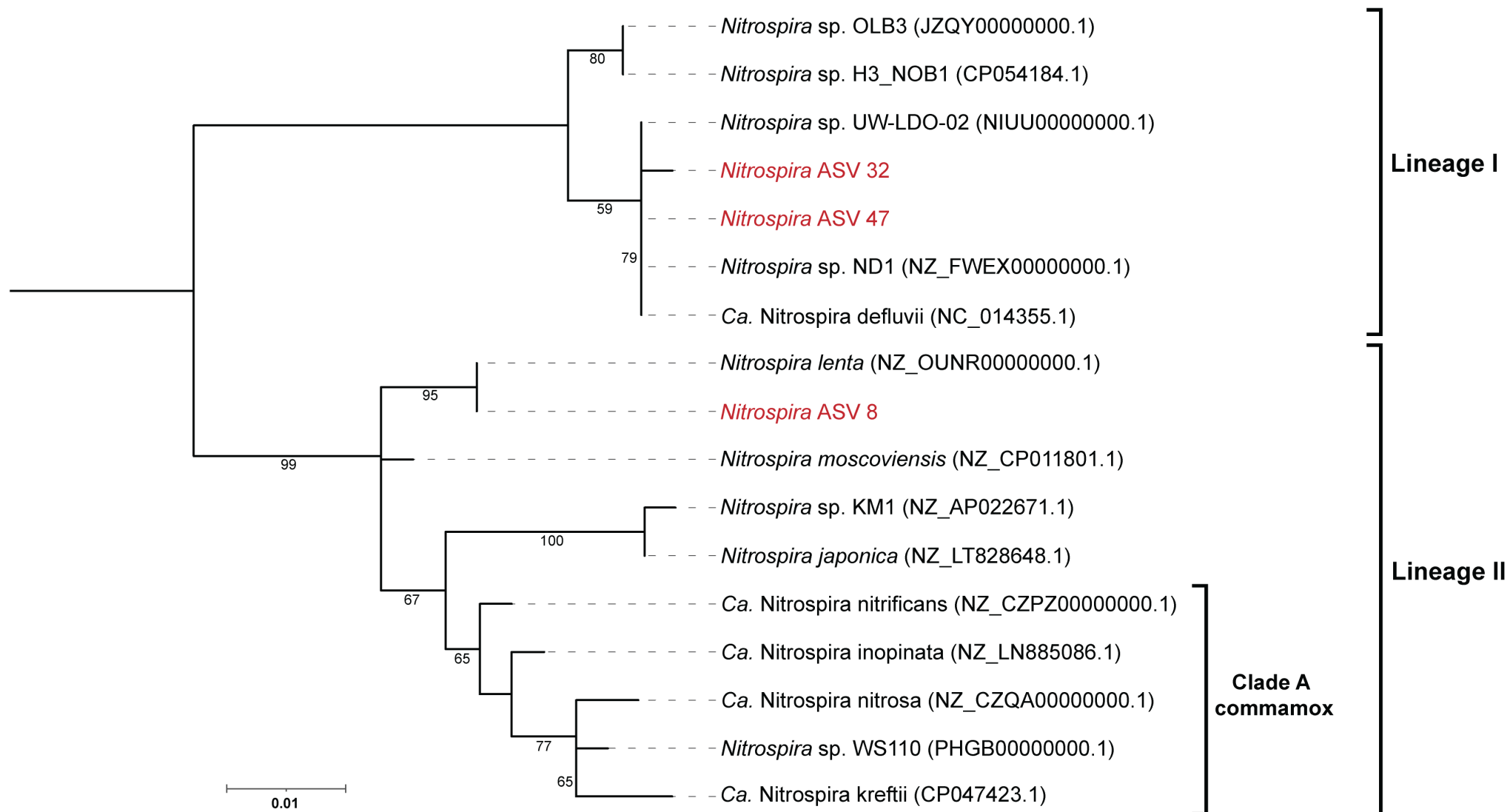

Supplement: FIG S7 [file msystems.00712-21-sf007.pdf]

DESeq2 Normalized Read Counts

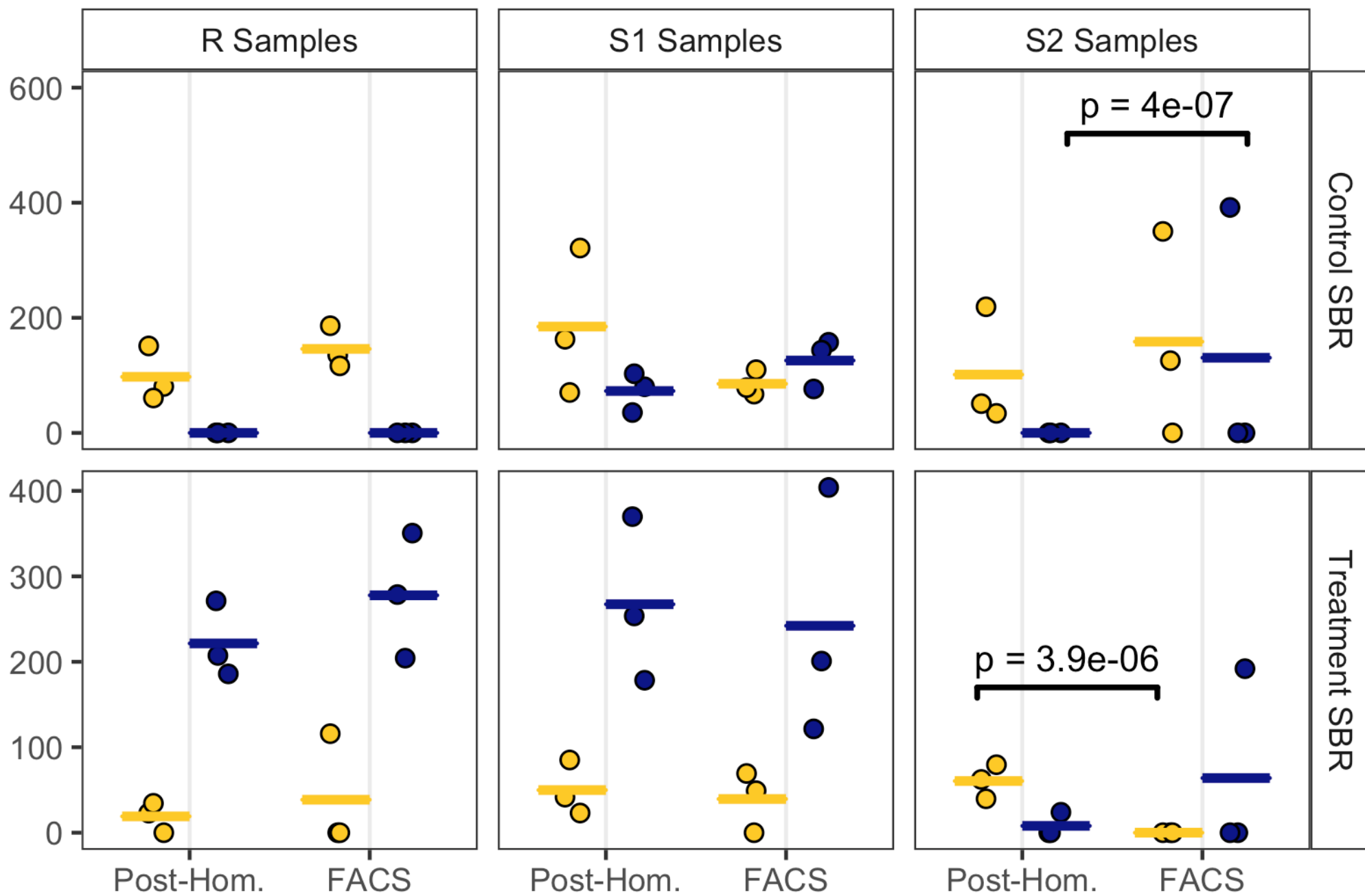

*Nitrosomonas* ASVs: ASV\_31 ASV\_36

Supplement: FIG S12 [file msystems.00712-21-sf012.pdf]

**A** R Samples

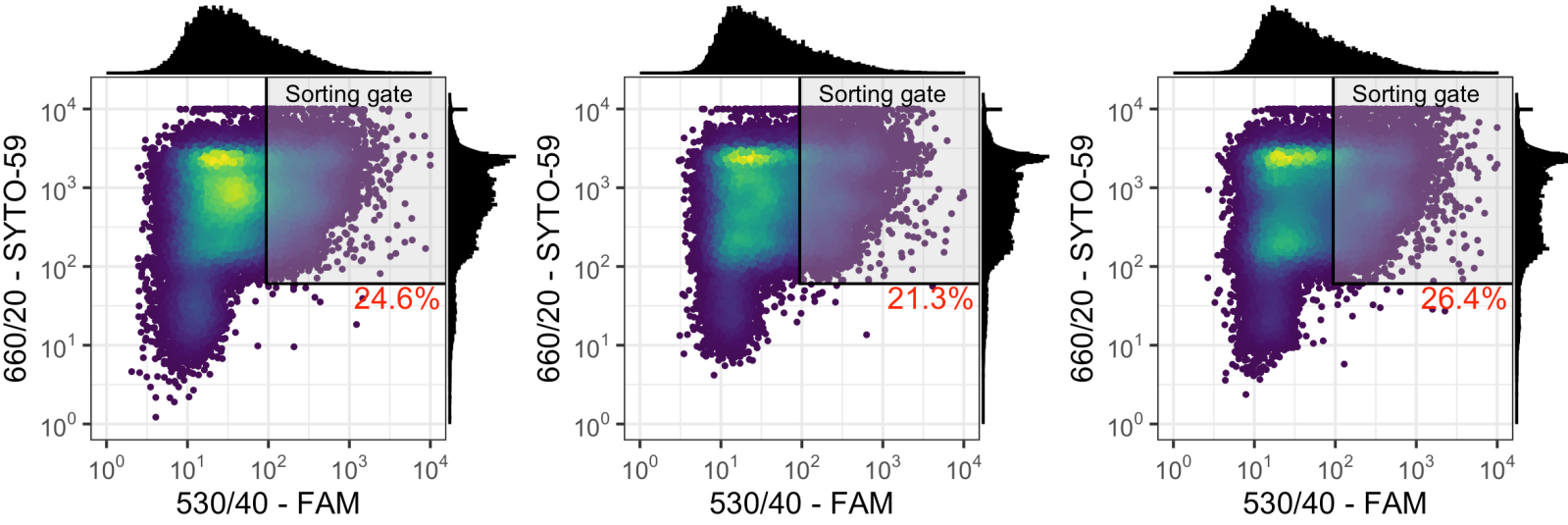

**B** S1 Samples

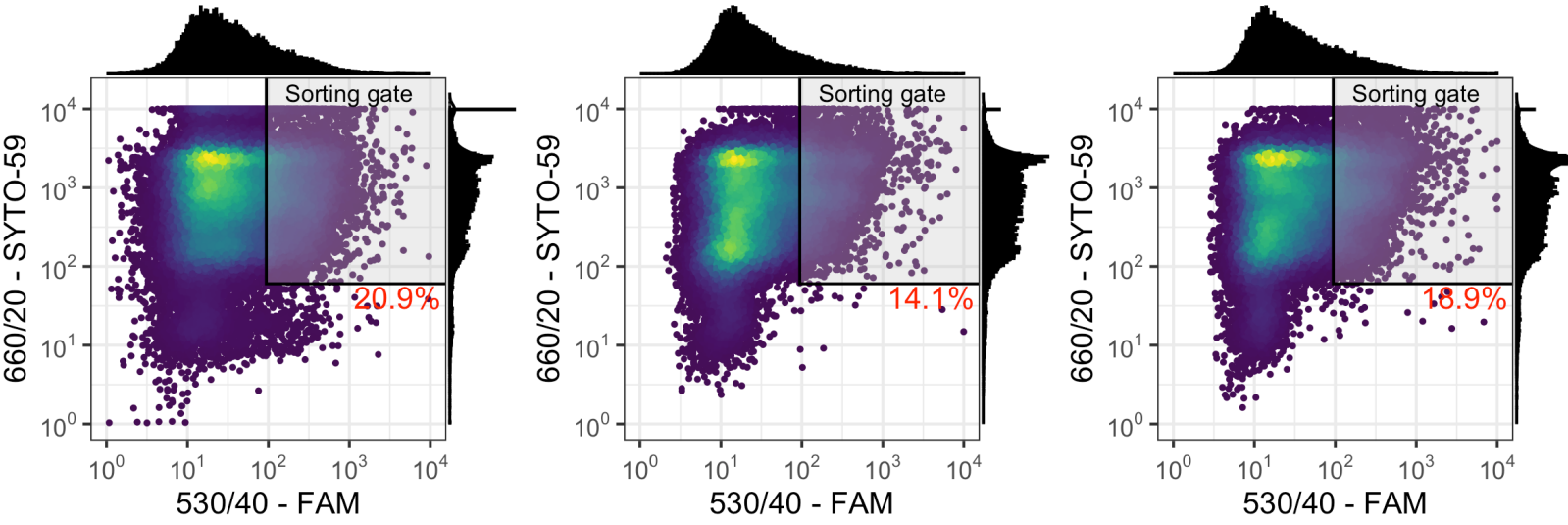

**C** S2 Samples

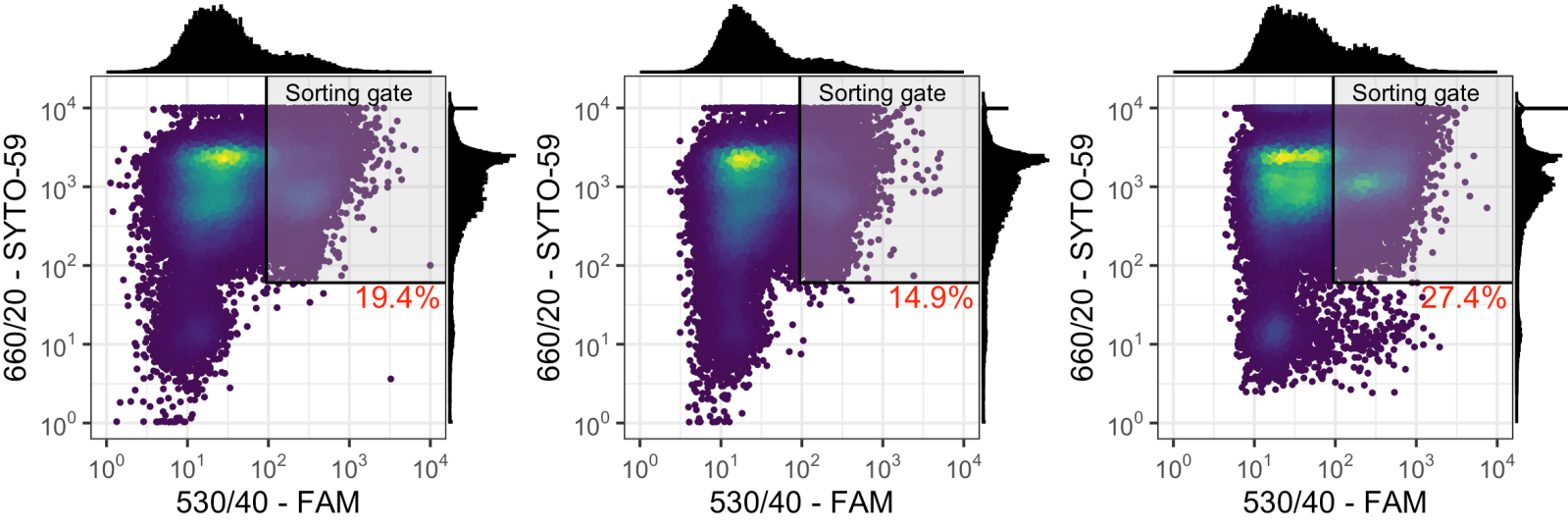

Supplement: FIG S9 [file msystems.00712-21-sf009.pdf]

**A** R Samples

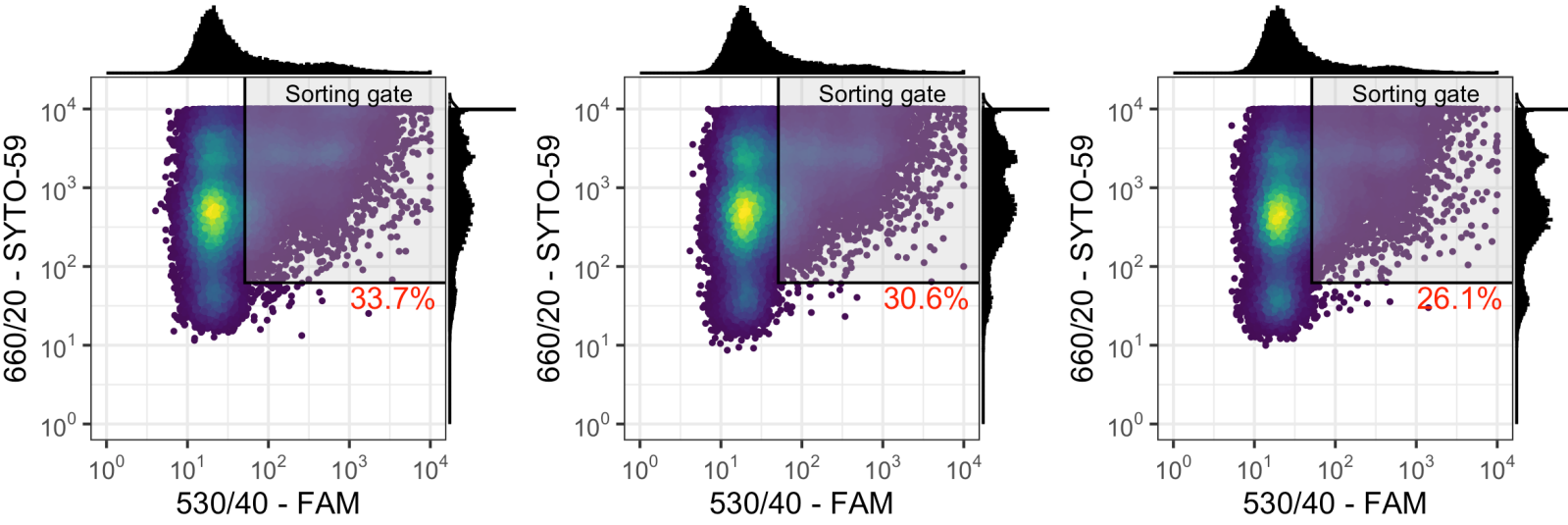

**B** S1 Samples

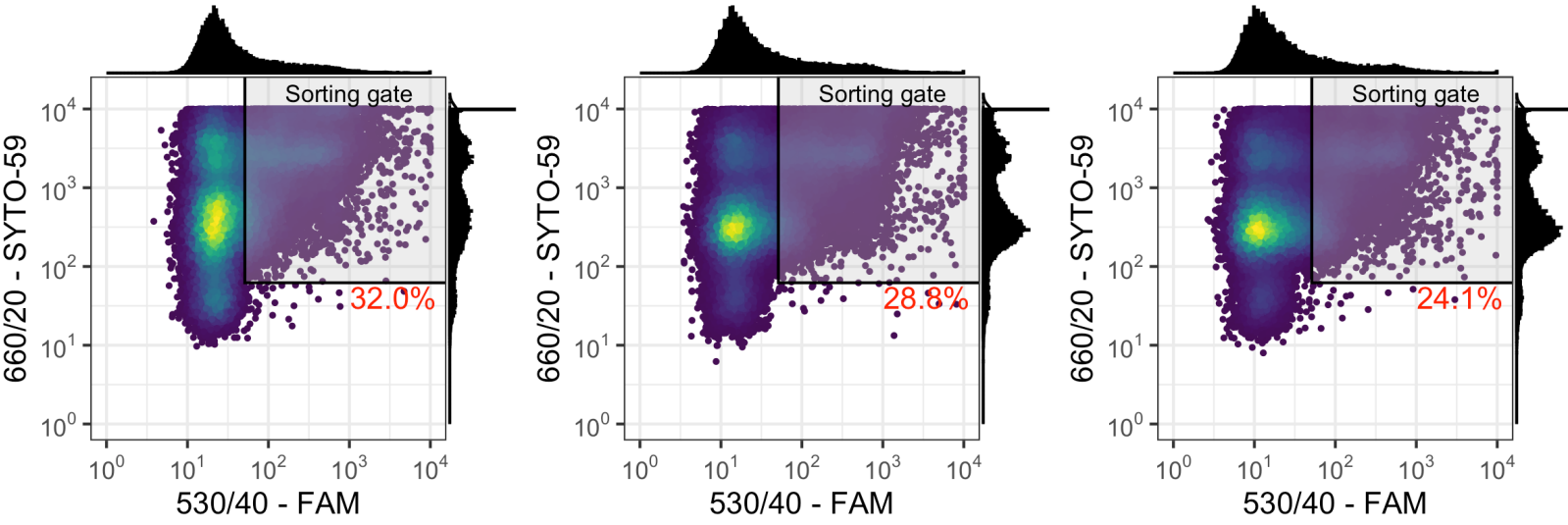

**C** S2 Samples

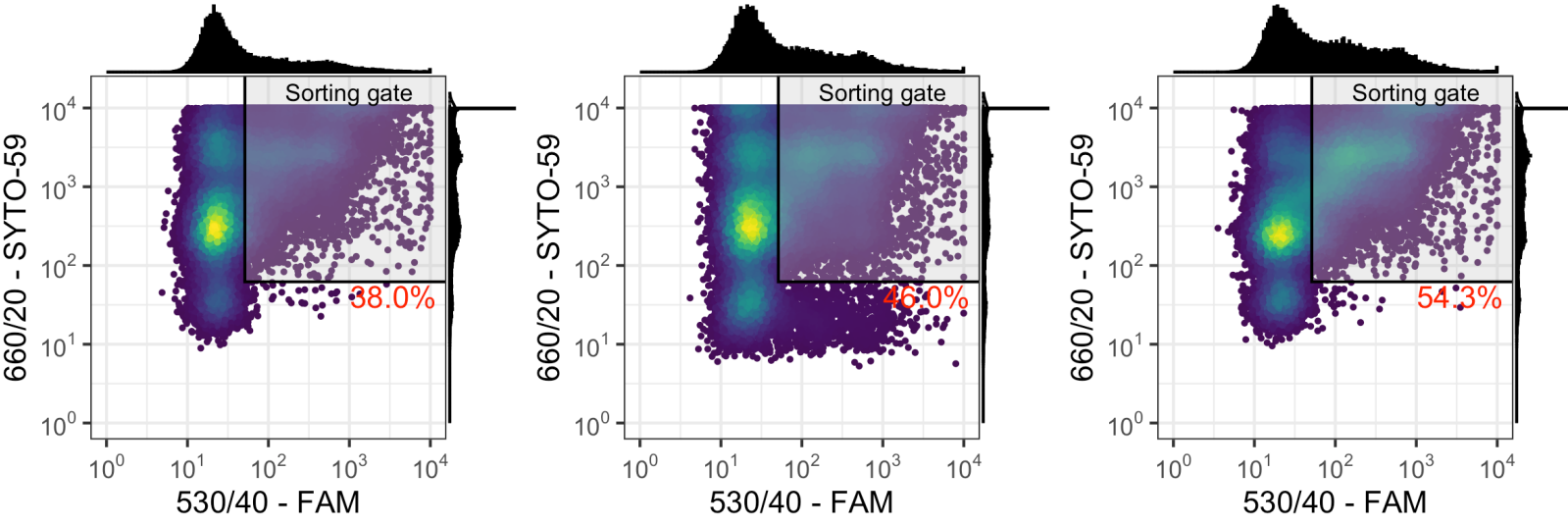

Supplement: FIG S10 [file msystems.00712-21-sf010.pdf]

**A** No-Stain Control

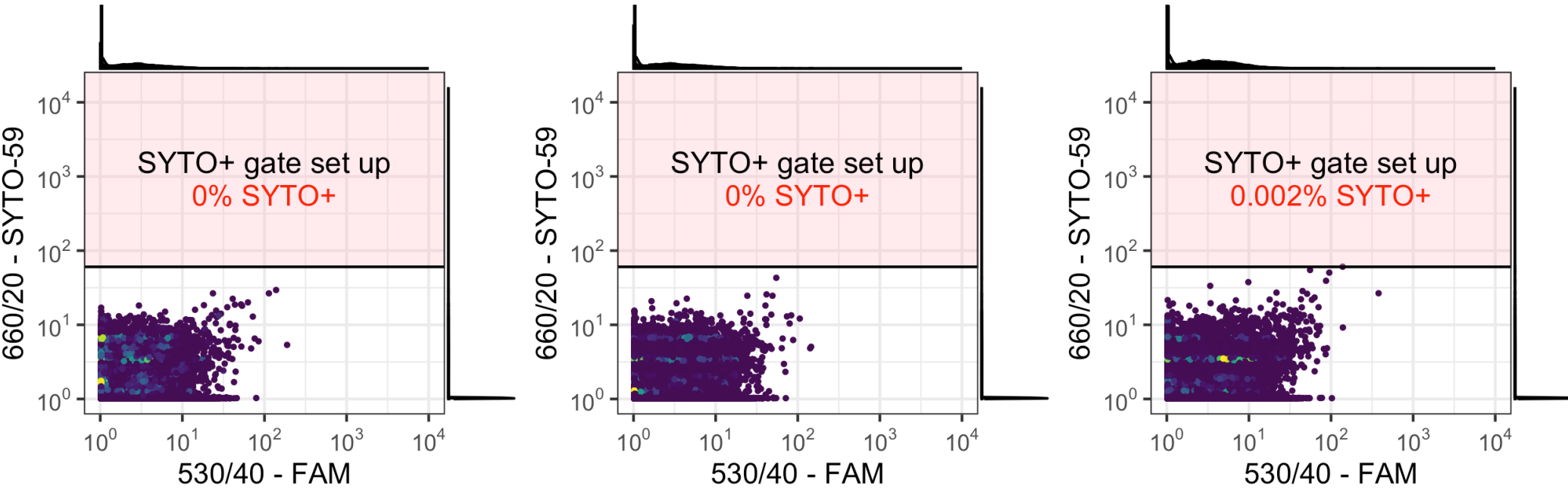

**B** HPG-Negative Control

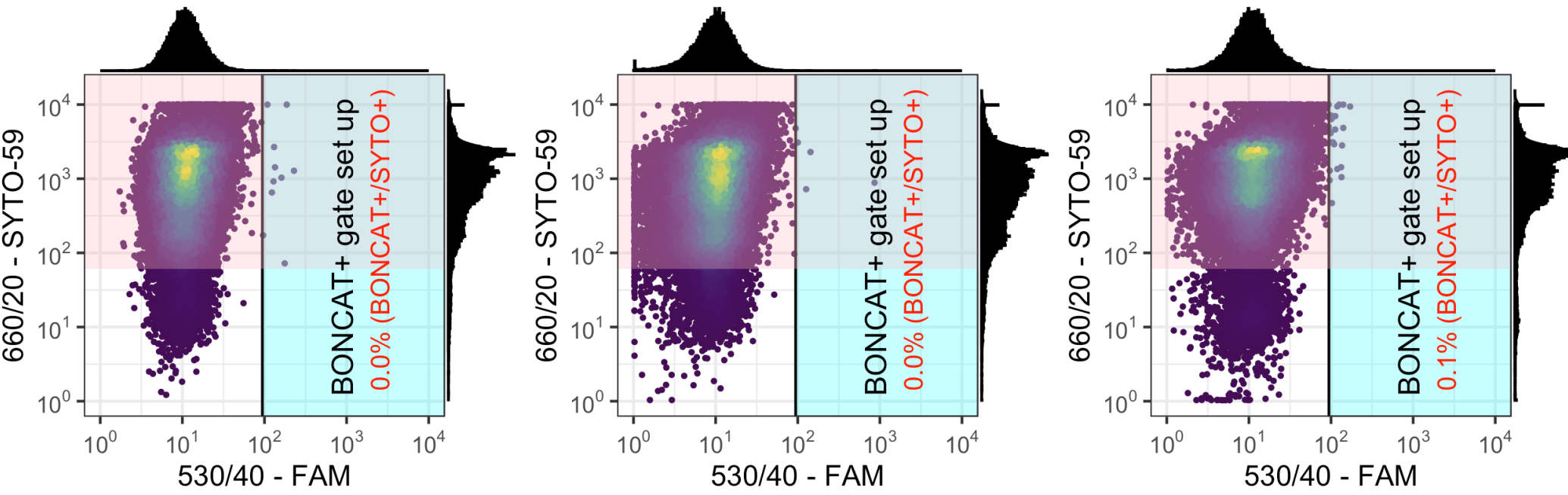

Supplement: FIG S13 [file msystems.00712-21-sf013.pdf]
