# Supplementary material for: Activity-Based Cell Sorting Reveals Resistance of Functionally Degenerate Nitrospira during a Press Disturbance in Nitrifying Activated Sludge
Source: mSystems. 2021 Jul 20;6(4):e00712-21. doi: 10.1128/mSystems.00712-21 (PMC8407113; doi:10.1128/mSystems.00712-21)
Supplement: TEXT S1 [file msystems.00712-21-s0001.pdf]

Text S1: Supplemental Methods

Micro and macro ingredients of synthetic wastewater:

The synthetic wastewater contained macro elements (162.4 mg/L of $MgCl_2$, 128.65 mg/L of $CaCl_2 \cdot 2H_2O$, 79.1 mg/L of KCl), trace elements (1.03 mg/L of $FeSO_4 \cdot 7H_2O$, 0.32 mg/L of $ZnSO_4 \cdot 7H_2O$, 0.055 mg/L of $CuSO_4 \cdot 5H_2O$, 0.0562 mg/L of $CoCl_2 \cdot 6H_2O$, 0.032 mg/L of $Na_2MoO_4 \cdot 2H_2O$, 0.05 mg/L of H_3BO_3, 0.05 mg/L of KI, 0.022 mg/L of $NiCl \cdot 6H_2O$, 0.14 mg/L of $Al_2(SO_4)_3 \cdot 18H_2O$, 0.283 mg/L of $MnCl_2 \cdot 4H_2O$, 6.87 mg/L of EDTA), phosphate (51.3 mg/L of $Na_2HPO_4 \cdot 7H_2O$, 16.7 mg/L of KH_2PO_4), alkalinity (298.3 mg/L of $NaHCO_3$), and yeast (20 mg/L).

Daily effluent monitoring experiments to determine composite nitrite accumulation ratios:

Monitoring experiments lasting 24 h were performed approximately every 10 days during the Treatment Phase to monitor daily nitrification performance over 8 of the 3 h SBR cycles. Mixed liquor samples were collected just after the addition of treated return sludge during the feeding period of the first cycle, and effluent samples were obtained during the settling period of the 1st, 2nd, 3rd, 4th, and 8th cycles thereafter. The nitrite accumulation ratio (NAR) of each cycle was calculated based on Equation 1, where S_{NO2} and S_{NO3} are the effluent nitrite and nitrate concentrations (mg-N/L), respectively.

Eqn. 1 $$NAR = S_{NO2} \div (S_{NO2} + S_{NO3})$$

Procedure and analysis of preliminary BONCAT validation microcosms:

Three preliminary BONCAT microcosms were conducted prior to the experimental microcosms to validate the sensitivity of BONCAT in labelling only active cells that had incorporated HPG. The BONCAT validation microcosms included: (1) an HPG-negative control; (2) a pre-incubation fixed control; and (3) a post-incubation fixed control. All validation microcosms were seeded with mixed liquor from the control SBR and conducted exactly as the experimental microcosms except with

the following modifications. For the pre-incubation fixed control, mixed liquor was first fixed with 3% paraformaldehyde for 1 hr at RT and washed three times in 1x PBS prior to incubation with HPG. For the post-incubation fixed control, unfixed mixed liquor was first incubated with HPG before undergoing the same paraformaldehyde fixation and PBS washing steps. The HPG-negative control microcosm was conducted exactly as those in the experimental microcosms. Samples from each validation microcosm were washed three times in 1x PBS, resuspended in 50% ethanol in 1x PBS (vol/vol) and stored at -20°C prior to undergoing identical sample preparation and FACS analysis as the experimental microcosm samples.

Homogenization procedure for BONCAT microcosm samples:

The enzymatic homogenization procedure described below was optimized to enable the extraction of single microbial cells from complex activated sludge floc with minimal cell destruction. An 800 µL volume from each microcosm sample was transferred to a 15 mL Falcon tube, washed with 4mL of 1x PBS and resuspended into 2 mL of a filter-sterilized enzymatic homogenization solution consisting of 1 mg/mL protease (Protease from Streptomyces griseus, Type XIV, >3.5 units/mg, MilliporeSigma, USA) and 0.25 mg/mL **α**-amylase (Alpha-amylase from Bacillus sp., ~380 U/mg, MilliporeSigma) in 1x PBS. Samples were incubated in a temperature-controlled chamber at 37 °C with 200 rpm orbital shaking for 1.5 h. Samples were taken from the chamber and vortexed for 20 s at maximum speed every 0.5 h (three times total) throughout the incubation period. After the incubation period, 8 mL of a cold (4 °C) filter-sterilized solution of 10 mM EDTA in 1x PBS was added to each sample before incubating samples on ice for 15 min. Samples were then sonicated in an ice-filled sonicating water bath for three 1 min bursts with a 30 s rest period between each burst. Samples were stored in an ice-bath prior to filter-immobilization and click-chemistry labeling (conducted within 1 h).

Click-chemistry labeling of homogenized BONCAT microcosm samples:

Filter-immobilized click-chemistry was performed closely following the procedure outlined by Couradeau et al. (6) and in general adherence to the procedure outlined by Hatzenpichler and Orphan(7). Multiple aliquots of the click-solution were prepared by mixing the dye premix with the reaction buffer (6, 7). Aliquots of the dye-premix were prepared fresh by mixing 1.25 uL of 20 mM copper sulfate ($CuSO_4$, 100 uM final concentration), 2.50 µL of 50 mM tris-hydroxypropyltriazolylmethylamine (THPTA, 500 uM final concentration) and 1.67 µL of 1.5 mM FAM picolyl azide dye (10uM final concentration; Click Chemistry Tools, USA) and allowing the mixture to react for 3 min at RT in the dark. Aliquots of the reaction buffer were prepared by adding 12.5 µL of freshly prepared 100 mM sodium ascorbate in 1x PBS (5mM final concentration) and 12.5 µL of freshly prepared 100 mM aminoguanidine HCl in 1x PBS (5 mM final concentration) to 221 µL of 1x PBS. The dye-premix was added to the reaction buffer and the solution mixed gently by inversion to create the 'click-solution'.

Using a sterile syringe filtration apparatus, each homogenized microcosm sample (800 µL original volume) was immobilized onto a 0.22 um GTTP Isopore Membrane filter (MilliporeSigma, USA) and washed with 20 mL of filter-sterilized 1x PBS by passage through the filtration apparatus. Filters were transferred onto glass slides and stored at RT in a light-shielded environment while the click-chemistry reaction mixture (click-solution) was prepared. Each filter-immobilized sample was covered with 80 µL of click-solution followed by a glass coverslip, and all samples were incubated in the dark for 30 min. Filter-immobilized samples were then washed three times in a series of 20 mL baths of 1x PBS for 5 min each to remove residual FAM dye. The washed filters were transferred into 15 mL Falcon tubes containing 2 mL of a filter-sterilized modified TE buffer (10mM Tris-Base, 5mM EDTA, pH 8) and three 2.3 mm zirconium/silica beads (11079125z, BioSpec, USA), and vortexed at 1800 rpm for 3.5 min to detach the cells from the filters. Each

sample was filtered through a 30 µm Nylon Mesh filter (Nylon Net Filter, 30.0 um pore size, Millipore) using a sterile syringe filtration apparatus prior to SYTO59 counterstaining and FACS analysis (FACS conducted within 3 h of sample preparation). The No-stain control samples were prepared from HPG-negative microcosm samples using the same procedure but were omitted from click-chemistry labeling (no click-solution added) and SYTO59 counterstaining. Epifluorescence imaging was conducted to validate the efficacy of the sample homogenization and click-chemistry labeling procedures, and to provide visual confirmation of HPG-dependent BONCAT labeling (data not shown).

Gating procedure for fluorescence activated cell sorting (FACS) of BONCAT-positive cells:

Prepared microcosm samples from each SBR were sorted in separate trials, with gates set independently in each trial to account for differences in biomass characteristics between the two SBRs. Initial gating was conducted using a gate drawn based on forward scatter and side scatter, followed by a gate drawn based on side scatter and trigger pulse width, to exclude large particles and cell aggregates. Sorting gates were established to simultaneously target SYTO59-positive (SYTO+) cells that were also BONCAT-positive (BONCAT+), as determined based on their FAM picolyl azide BONCAT dye fluorescence. A first gate was drawn to target STYO+ cells from background particles by analyzing the background SYTO59 fluorescence of cells in triplicate No-stain control samples (no click-chemistry, no SYTO59 counterstaining) and setting the gate such that $< 0.5\%$ false positives were included (Figures S13 and S14). A second gate was drawn to target BONCAT+ cells from non-HPG labelled cells by analyzing the background FAM fluorescence in triplicate HPG-negative control samples that underwent click-chemistry labeling and SYTO59 counterstaining, and setting the gate such that $< 0.5\%$ false positives were included (Figures S13 and S14). The sorting gate was then drawn as the area of overlap between the SYTO+ and BONCAT+ gates. The triplicate pre-sort samples from each HPG-positive microcosm

were sorted based on their SYTO59 and FAM fluorescence using this predetermined sorting gate, where the fraction of BONCAT+ cells in each sample was calculated as the fraction of SYTO+ cells residing in the sorting gate.

Low-biomass input DNA extraction procedure for BONCAT microcosm samples:

The pre-homogenized, pre-sort, BONCAT-FACS samples prepared from each BONCAT microcosm were all extracted using the PrepGEM Bacteria kit (ZyGEM, New Zealand) following a procedure that was optimized for the extraction of the low biomass input BONCAT-FACS samples (i.e. low-biomass input procedure). The low-biomass input extraction procedure is outlined as follows. Samples were pulled from -80 ^{o}C storage and thawed on ice at 4 ^{o}C overnight. To each sample, 900 uL of ice-cold (-18 ^{o}C) 100% ethanol and 5.4 uL of 5mg/mL linear acrylamide (ThermoFisher Scientific, USA) were added before mixing samples by vortexing and incubating samples at -18 ^{o}C for 2 h to precipitate any DNA that may have been released by cell lysis during sample storage. The samples were then centrifuged (20,000 x g, 4 ^{o}C, 40 min) and the supernatant was removed through careful pipetting. Sample tubes were then opened and incubated in a heat block for 10 min at 37 ^{o}C to remove any residual supernatant through evaporation. A bulk volume of extraction mix was prepared following the prepGEM Bacteria kit instructions, and 10ul of the extraction mix was added to each sample before mixing samples briefly by vortexing. Extractions were then conducted by incubating samples in a heat block for 15 min at 37°C, 10 min at 75°C, and 2min at 92°C. The extracted samples were stored at 4°C prior to 16S rRNA gene amplification and sequencing.

Estimating active biomass on VSS basis:

Steady-state mass-balance equations for complete-mix AS with sludge recycle were used to predict the fraction of reactor VSS that was active biomass (X_{bio}) (8):

$$X_{bio} = X_H + X_{AOB} + X_{NOB}$$

$$X_{bio} = (SRT/HRT)[(Y_H)(COD_r)/(1 + b_H SRT) + (Y_{AOB})(NH_{4,ox})/(1 + b_{AOB} SRT) + (Y_{NOB})(NH_{4,ox})(1 - NAR)/(1 + b_{NOB} SRT)]$$

where:

SRT = solids retention time (10 d)

HRT = hydraulic retention time (0.5 d)

Y_H= heterotrophic yield (0.4 g VSS/g COD) (8)

b_H= heterotrophic decay constant (0.12 d^{-1}) (8)

COD_r = COD removed (100 mg/L)

Y_{AOB} = AOB yield (0.15 g VSS/g N) (8)

b_{AOB}= AOB decay constant (0.17 d^{-1}) (8)

$NH_{4,ox}$= amount of ammonium oxidized (~85% of influent (8) = 43 mg N/L flow-weighted average in FA phase)

Y_{NOB} = NOB yield (0.05 g VSS/g N) (8)

b_{NOB}= NOB decay constant (0.17 d^{-1}) (8)

NAR= nitrite accumulation ratio ($NO_2^-/(NO_3^-+NO_2^-)$), used to infer fraction of un-oxidized NO_2-N (11% in Treatment SBR, 1% in control SBR)

With the above parameters, X_{bio} is estimated to be 426 mg VSS/L and 427 in the Treatment and control SBRs, respectively, which account for 77% and 67% of the total reactor VSS, respectively (Figure S2). This analysis assumes the decay constants were similar in two reactors, which may not have been the case.

Analytical methods for reactor monitoring:

Please see Supplemental Text Table 1 for a summary of analytical methods used for bioreactor monitoring.

Bioinformatics script for denoising 16S rRNA gene amplicon data with DADA2:

The following markdown was run in the R environment (v.3.6.1). The code chunks in grey background were run for this analysis.

###1. Load libraries

```
library(dada2); packageVersion("dada2")
```

```
library(tidyverse);
```

In this study, we had 2 different MiSeq runs on amplicons targeting the V4-V5 region of the 16S rRNA gene. Because error rates may be different for each run, we will do denoising, and merging on a run-by-run basis. But, to ensure the same amplicon size, we will filter all reads together.

###2. Quality filter reads

Is this R-markdown being run for the first time, or would you like to override previously saved data?

```
ovr <- "FALSE" #change to TRUE if you want to override data
```

Set path directory to where raw_seqs folder is stored

```
#Import file names
path <- "./16S_iTags" # CHANGE ME to the directory containing the analysis files.
path_fs <- paste0(path,"/raw_seqs")
```

Read in sample metadata:

```
sampledata_pn <- read.csv(file=paste0(path,"/metadata.csv"), header=TRUE, row.names=1, stringsAsFactors = FALSE) %>% #read in sample metadata from plate submission to sequencing facility
  dplyr::filter(Reactor == "R1" | Reactor =="R2") %>% #filter metadata to just select PN samples
  mutate(Sample = paste0(Plate_Column, Plate_Row)) %>% #use plate row and column to make sample names like the .fastq files
    mutate(Sample = str_replace_all(Sample, "-", "_"))
tail(sampledata_pn)
```

Read in file names

```
fns <- list.files(path_fs)
fns
fastqs <- fns[grepl("001.fastq$", fns)]
fastqs <- sort(fastqs) # Sort ensures forward/reverse reads are in same order
fnFs <- fastqs[grepl("_R1", fastqs)] # Just the forward read files
fnRs <- fastqs[grepl("_R2", fastqs)] # Just the reverse read files
```

Fully specify the path for the fnFs and fnRs

```
fnFs <- file.path(path_fs, fnFs)
fnFs
fnRs <- file.path(path_fs, fnRs)
```

3. Filter and trim reads

Visualizing the quality profiles along the sequencing reads

```
plotQualityProfile(fnFs[[1]])
plotQualityProfile(fnRs[[1]])
```

Make directory and filenames for the filtered fastqs:

```
filt_path <- file.path(path, "filtered")
if(!file_test("-d", filt_path)) dir.create(filt_path)
if(ovr) filtFs <- file.path(filt_path, paste0(sample.names, "_F_filt.fastq.gz")) #create placeholder files for filtered reads
if(ovr) filtRs <- file.path(filt_path, paste0(sample.names, "_R_filt.fastq.gz"))
names(filtFs) <- sample.names
names(filtRs) <- sample.names
```

Filter sequences by trimming first 10bp, truncating F reads at 275bp and R reads at 225bp

```
if(ovr) out <- filterAndTrim(fnFs, filtFs, fnRs, filtRs, trimLeft=c(15, 15), truncLen=c(275,225),maxN=0, maxEE=c(2,2), truncQ=2, rm.phix=TRUE,compress=TRUE, multithread=TRUE)
# On Windows set multithread=FALSE
if(ovr) saveRDS(out, file=paste0(path,"/out.RDS")) #save R object in case we need to re-run later
if(file_test("-f", paste0(path,"/out.RDS"))) out <- readRDS(file=paste0(path,"/out.RDS"))
```

Learn error rates of forward reads for run 1

```
if(ovr) errF1 <- learnErrors(filtFs1, multithread=TRUE)
if(ovr) saveRDS(errF1, file=paste0(path,"/errF1.RDS")) #save R object in case we need to re-run later
if(file_test("-f", paste0(path,"/errF1.RDS"))) errF1 <- readRDS(file=paste0(path,"/errF1.RDS"))
```

Learn error rates of reverse reads for run 1

```
if(ovr) errR1 <- learnErrors(filtRs1, multithread=TRUE)
if(ovr) saveRDS(errR1, file=paste0(path,"/errR1.RDS")) #save R object in case we need to re-run later
if(file_test("-f", paste0(path,"/errR1.RDS"))) errR1 <- readRDS(file=paste0(path,"/errR1.RDS"))
```

Learn error rates of forward reads for run 2

```
if(ovr) errF2 <- learnErrors(filtFs2, multithread=TRUE)
if(ovr) saveRDS(errF2, file=paste0(path,"/errF2.RDS")) #save R object in case we need to re-run later
if(file_test("-f", paste0(path,"/errF2.RDS"))) errF2 <- readRDS(file=paste0(path,"/errF2.RDS"))
```

Learn error rates of reverse reads for run 2

```
if(ovr) errR2 <- learnErrors(filtRs2, multithread=TRUE)
if(ovr) saveRDS(errR2, file=paste0(path,"/errR2.RDS")) #save R object in case we need to re-run later
if(file_test("-f", paste0(path,"/errR2.RDS"))) errR2 <- readRDS(file=paste0(path,"/errR2.RDS"))
```

Plot errors

```
plotErrors(errF1, nominalQ=TRUE)
```

```
plotErrors(errF2, nominalQ=TRUE)
plotErrors(errR1, nominalQ=TRUE)
plotErrors(errR2, nominalQ=TRUE)
```

Sample inference using above model fit, run1

```
#Forward reads
if(ovr) dadaFs1 <- dada(filtFs1, err=errF1, multithread=TRUE)
if(ovr) saveRDS(dadaFs1, file=paste0(path,"/dadaFs1.RDS")) #save R object in case we need to re-run later
if(file_test("-f", paste0(path,"/dadaFs1.RDS"))) dadaFs1 <- readRDS(file=paste0(path,"/dadaFs1.RDS"))

#Reverse reads
if(ovr) dadaRs1 <- dada(filtRs1, err=errR1, multithread=TRUE)
if(ovr) saveRDS(dadaRs1, file=paste0(path,"/dadaRs1.RDS")) #save R object in case we need to re-run later
if(file_test("-f", paste0(path,"/dadaRs1.RDS"))) dadaRs1 <- readRDS(file=paste0(path,"/dadaRs1.RDS"))
```

Sample inference using above model fit, run2

```
#Forward reads
if(ovr) dadaFs2 <- dada(filtFs2, err=errF2, multithread=TRUE)
if(ovr) saveRDS(dadaFs2, file=paste0(path,"/dadaFs2.RDS")) #save R object in case we need to re-run later
if(file_test("-f", paste0(path,"/dadaFs2.RDS"))) dadaFs2 <- readRDS(file=paste0(path,"/dadaFs2.RDS"))

#Reverse reads
if(ovr) dadaRs2 <- dada(filtRs2, err=errR2, multithread=TRUE)
if(ovr) saveRDS(dadaRs2, file=paste0(path,"/dadaRs2.RDS")) #save R object in case we need to re-run later
if(file_test("-f", paste0(path,"/dadaRs2.RDS"))) dadaRs2 <- readRDS(file=paste0(path,"/dadaRs2.RDS"))
```

4. Merge paired reads

Check that samples are in right order

```
if(!identical(names(filtFs1), names(filtRs1))) stop("Forward and reverse files do not match.")
if(!identical(names(filtFs2), names(filtRs2))) stop("Forward and reverse files do not match.")
```

Run 1

```
if(ovr) mergers1 <- mergePairs(dadaFs1, filtFs1, dadaRs1, filtRs1, verbose=TRUE)
```

Run 2

```
if(ovr) mergers2 <- mergePairs(dadaFs2, filtFs2, dadaRs2, filtRs2, verbose=TRUE)
```

Make sequence tables:

Run 1

```
if(ovr) seqtab1 <- makeSequenceTable(mergers1)
if(ovr) saveRDS(seqtab1, file=paste0(path,"/seqtab1.RDS")) #save R object in case we need to re-run later
```

```
if(file_test("-f",     paste0(path,"/seqtab1.RDS")))     seqtab1     <-
readRDS(file=paste0(path,"/seqtab1.RDS"))
dim(seqtab1)
```

Run 2

```
if(ovr) seqtab2 <- makeSequenceTable(mergers2)
if(ovr)  saveRDS(seqtab2,  file=paste0(path,"/seqtab2.RDS"))  #save  R
object in case we need to re-run later
if(file_test("-f",     paste0(path,"/seqtab2.RDS")))     seqtab2     <-
readRDS(file=paste0(path,"/seqtab2.RDS"))
dim(seqtab2)
```

5. Remove chimeras

```
if(ovr) seqtab <- mergeSequenceTables(seqtab1, seqtab2)
if(ovr) saveRDS(seqtab, file=paste0(path,"/seqtab.RDS")) #save R object
in case we need to re-run later
if(file_test("-f",     paste0(path,"/seqtab.RDS")))     seqtab     <-
readRDS(file=paste0(path,"/seqtab.RDS"))

if(ovr) seqtab.nochim <- removeBimeraDenovo(seqtab, method="consensus",
multithread=TRUE, verbose=TRUE)
if(ovr) saveRDS(seqtab.nochim, file=paste0(path,"/seqtab_nochim.rds"))
if(file_test("-f", paste0(path,"/seqtab_nochim.rds"))) seqtab.nochim <-
readRDS(file=paste0(path,"/seqtab_nochim.rds"))
```

Supplemental Text Table S1: Summary of analytical methods used for monitoring bioreactors

Analyte	Method	Sample Pretreatment	Instrument
TSS	Standard Method 2540 D. Total Suspended Solids Dried at 103–105°C	Filtered onto glass microfiber filters (0.45-μm pore size, Whatman, Pittsburgh, USA), and dried in oven for 12 h	Fisher Scientific Isotemp 737F Oven for drying; Ohaus Adventurer AR2140 Analytical Balance (Ohaus, Parsippany, NJ, USA) for weighing
VSS	Standard Method 2540 E. Fixed and Volatile Solids Ignited at 550°C	Ignite residue from TSS measurement in furnace for 30 min	Thermolyne 30400 Furnace (Thermo Fisher Scientific) for burning; Ohaus Adventurer AR2140 Analytical Balance (Ohaus, Parsippany, NJ, USA) for weighting
Ammonia (NH_3-N)	Standard Method 4500-NH_3 H. Flow Injection Analysis	Centrifuged at 5000 rpm for 5 min, filtered through glass filters (0.2-μm pore size, Fisherbrand™), and stored at 4 °C before measurement	Thermo Iec Multi Rf Centrifuge (Thermo IEC, USA); Lachat QuikChem 8000 Series (Lachat Instrument, Wisconsin)
Nitrite (NO_2^--N)	Standard Method 4500-NO_2^- I. Cadmium Reduction Flow Injection Method	Centrifuged at 5000 rpm for 5 min, filtered through glass filters (0.2-μm pore size, Fisherbrand™), and stored at 4 °C before measurement	Thermo Iec Multi Rf Centrifuge (Thermo IEC, USA); Lachat QuikChem 8000 Series (Lachat Instrument, Wisconsin)
Nitrate (NO_3^--N)	Standard Method 4500-NO_3^- I. Cadmium Reduction Flow Injection Method	Centrifuged at 5000 rpm for 5 min, filtered through glass filters (0.2-μm pore size, Fisherbrand™), and stored at 4 °C before measurement	Thermo Iec Multi Rf Centrifuge (Thermo IEC, USA); Lachat QuikChem 8000 Series (Lachat Instrument, Wisconsin)
Orthophosphate (PO_4^{3-}-P)	Standard Method 4500-P G. Flow Injection Analysis for Orthophosphate	Centrifuged at 5000 rpm for 5 min, filtered through glass filters (0.2-μm pore size, Fisherbrand™), and stored at 4 °C before measurement	Thermo Iec Multi Rf Centrifuge (Thermo IEC, USA); Lachat QuikChem 8000 Series (Lachat Instrument, Wisconsin)

sCOD	N/A	Centrifuged at 5000 rpm for 5 min, filtered through glass filters (0.2-µm pore size, Fisherbrand™), acidified to pH 2 by H_2SO_4, and stored at 4 ºC before measurement	Thermo Iec Multi Rf Centrifuge (Thermo IEC, USA); HP 6890 Series GC system (Hewlett Packard (Agilent))
pH	N/A	Take less than 10 mL of mixed liquor from SBRs into beakers	Beckman 40 pH Meter (Beckman Coulter, Massachusetts, USA)
DO	N/A	N/A	HQ30D Portable DO Meter (Hach, Colorado, USA)

References:

1. Seemann T. barrnap 0.9: Rapid Ribosomal RNA Prediction. https://github.com/tseemann/barrnap
2. Edgar RC. 2004. MUSCLE: multiple sequence alignment with high accuracy and high throughput. Nucleic Acids Res 32:1792–1797.
3. Kumar, Stecher, Li, Knyaz, and Tamura. 2018. MEGA X: Molecular Evolutionary Genetics Analysis across computing platforms. Molecular biology and evolution, 35(6), 1547.
4. Nguyen L-T, Schmidt HA, von Haeseler A, Minh BQ. 2015. IQ-TREE: A Fast and Effective Stochastic Algorithm for Estimating Maximum-Likelihood Phylogenies. Mol Biol Evol 32:268–274.
5. Letunic I, Bork P. 2021. Interactive Tree Of Life (iTOL) v5: an online tool for phylogenetic tree display and annotation. Nucleic Acids Res gkab301.
6. Couradeau E, Sasse J, Goudeau D, Nath N, Hazen TC, Bowen BP, Chakraborty R, Malmstrom RR, Northen TR. 2019. Probing the active fraction of soil microbiomes using BONCAT-FACS. Nat Commun 10:2770.
7. Hatzenpichler R, Orphan VJ. 2015. Detection of Protein-Synthesizing Microorganisms in the Environment via Bioorthogonal Noncanonical Amino Acid Tagging (BONCAT), p. 145–157. *In* McGenity, TJ, Timmis, KN, Nogales, B (eds.), Hydrocarbon and Lipid Microbiology Protocols. Springer Berlin Heidelberg, Berlin, Heidelberg.
8. Metcalf & Eddy, Inc., Tchobanoglous G, Burton F, Stensel HD. 2014. Wastewater Engineering: Treatment and Resource Recovery, 5th ed. McGraw-Hill.
